# Detecting signatures underlying the composition of biological data

**DOI:** 10.1093/nar/gkaf1388

**Published:** 2025-12-29

**Authors:** Anthony Duncan, Wing Koon, Katarzyna Sidorczuk, Christopher Quince, Clémence Frioux, Falk Hildebrand

**Affiliations:** Earlham Institute, Norwich Research Park, NR4 7UZ Norwich, United Kingdom; Quadram Institute Bioscience, Norwich Research Park, NR4 7UQ Norwich, United Kingdom; Earlham Institute, Norwich Research Park, NR4 7UZ Norwich, United Kingdom; Quadram Institute Bioscience, Norwich Research Park, NR4 7UQ Norwich, United Kingdom; Earlham Institute, Norwich Research Park, NR4 7UZ Norwich, United Kingdom; Quadram Institute Bioscience, Norwich Research Park, NR4 7UQ Norwich, United Kingdom; Earlham Institute, Norwich Research Park, NR4 7UZ Norwich, United Kingdom; Quadram Institute Bioscience, Norwich Research Park, NR4 7UQ Norwich, United Kingdom; Inria, University of Bordeaux, INRAE, 33400 Talence, France; Earlham Institute, Norwich Research Park, NR4 7UZ Norwich, United Kingdom; Quadram Institute Bioscience, Norwich Research Park, NR4 7UQ Norwich, United Kingdom; School of Biological Science, University of East Anglia, Norwich NR4 7TJ, United Kingdom

## Abstract

Biological compositional data is inherently multidimensional and therefore difficult to visualize and interpret. To allow for the automatic decomposition of large compositional data and to capture gradients in co-occurring features, called signatures, we developed a new software package ‘cvaNMF’. Our benchmarks on synthetic data show the effectiveness of cross-validation and our novel signature-similarity method to identify a suitable decomposition using non-negative matrix factorization (NMF). This software provides a complete set of tools to identify and visualize biologically informative signatures which we demonstrate in a wide range of microbial and cellular datasets: ‘Enterosignatures’ detected in gut metagenomes differentiated human hosts with diverse diseases; five ‘terrasignatures’ from rhizosphere metagenomes differentiated root- or soil-associated microbiomes, while being refined enough to infer geographic distances between plants. Large-scale data from >13 000 metagenomes representing 25 biomes were decomposed into environmental and host-associated microbiomes based on five newly discovered signatures. Finally, analysis of the cell composition of non-small cell lung cancer samples allowed separation of cancerous and inflamed tissues based on four cell-type signatures.

## Graphical Abstract

**Graphical Abstract:**
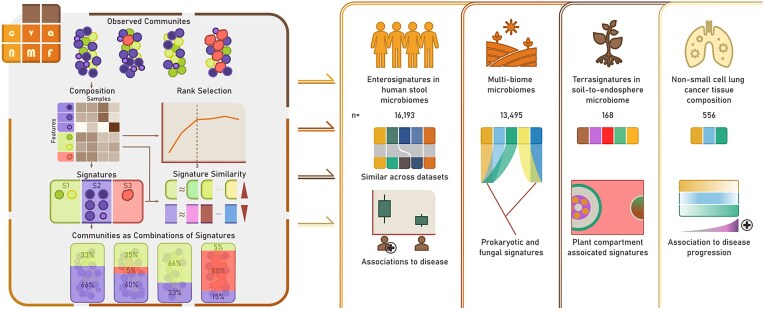

## Introduction

Microbial communities are found in a wide array of diverse ecological niches, from the seemingly remote such as the deep sea to those more familiar such as our skin and gut. The diversity and complexity of communities varies, sometimes consisting of a single bacterial species such as the bioluminescent symbiont of the bobtail squid light organ [1], but in most cases is much more complex, with even small samples of soils hosting an estimated tens of thousands of species [2]. Complex microbial communities can be subject to a wide range of variable abiotic conditions, whether moisture and nutrient levels in soil [3], or oxygen concentration and dietary intake in mammalian digestive systems [4–7], that rarely form discrete boundaries. Even starkly contrasting environments such as the sunlit and dark ocean have gradients in light levels as well as nutrients, temperature, and other conditions that will influence bacterial communities [8]. Observations along these gradients, or affected by multiple conditions, necessitate a description through gradual changes, requiring specialized clustering models that reflect mixed rather than discrete nature to provide a description which more intuitively reflects biological reality.

Several methods have been proposed to identify broad patterns in complex compositional data which describes the relative proportion of parts in a whole community, to determine biologically meaningful groupings of samples and/or features, with the aim to pinpoint shared biological similarity amongst clusters. In human gut metagenomes ‘Enterotypes’ (ET) were identified by clustering samples using a range of methods [9–13], with multiple studies independently finding clustering driven by *Bacteroides, Prevotella*, or *Firmicutes*. Similarly, some studies have grouped taxa to identify communities of commonly cooccurring microbes [11, 14, 15]. However, to simultaneously capture both clustering of samples and features, we recently used an unsupervised machine learning method, non-negative matrix factorization (NMF) [16], to produce a parts-based representation of the healthy human gut microbiome as a mixture of five ‘Enterosignatures’ (ES) [17]. NMF was chosen as it represents each sample as a linear mixture of several underlying signatures, where each signature is a group of multiple features (i.e. taxa), and each sample is a mixture of these signatures. These signatures represent potential gut bacterial guilds, taxa which show consistent co-abundance and complimentary function [18], and were both consistent with previously conducted hierarchical clustering (e.g. enterotypes [14, 19]), while representing different bacterial guilds as fractions of entire microbiomes, thereby enabling gradual shifts in Enterosignature composition and allowing for a highly resolved gut microbial analysis [17]. NMF has been frequently used in analysis of cancer data of various forms, with one of the earliest applications of NMF to biological data identifying leukaemia subtypes from gene expression data [20], and more recently to identify mutational signatures in cancer genomes [21], or cell-states from tumour single-cell transcriptomics [22]. In microbiomes, NMF has been applied to both marine microbiome function and ASV data [23, 24] at a relatively small scale (<100 samples), while in the human gut microbiome a constrained variant identified groups of fibre degradation genes [25] and supervised variants suggested a better separation of phenotypes [26].

To enable the wider useability of unsupervised NMF in data-driven biology, we provide a new python/nextflow pipeline ‘cvaNMF’ [shee·vah·en·em·ef]. The pipeline is optimized for large datasets and implements multiple methods for suggesting a suitable number of signatures. Our benchmark demonstrates how well established [20, 27] optimality criteria are outperformed by block bicrossvalidation using Gabriel-style holdouts [28, 29] or our novel ‘signature similarity’ measure. This standardized pipeline allowed us to more easily apply methods we previously used in analysis of the healthy gut microbiome to identify the typical microbial guilds found in soil, environmental, and dysbiotic gut microbiomes, characterizing meaningful guilds of bacteria with biological relevance, as well distinguishing groups of cells involved in inflammatory immune response and cancerous activity in a single cell cancer dataset, where cvaNMF identified signatures correlated to disease stage.

## Materials and methods

### Bicrossvalidation definition

Input matrix $X$ is split into $mn$ blocks being split evenly $m$ times along rows and $n$ times along columns. These blocks can be arranged into the structure shown in Supplementary Fig. S17. During the bicrossvalidation process, one block $A$ will be held out and an estimate $A\text{'}$ calculated using the remaining blocks. The suitability of the rank is measured by how well $A\text{'}$ approximates $A$. Each of the $mn$ blocks will be used once as the hold out $A$.

We use term $mn$-fold as a shorthand to refer to the design, so 9-fold bicrossvalidation divides the input into 9 blocks. We used a 3 × 3, 9-fold design throughout this work, and this is also the default in the package though other designs can be specified.

Using the labelling of matrices from Supplementary Fig. S17, bicrossvalidation is carried out as in [17, 29] using the following steps.

1.
$D$ is decomposed to ${{W}_D}$, ${{H}_D}$ using NMF.
2.
$B$ is decomposed with the $H$ matrix initialized as ${{H}_D}$ and not updated, to obtain ${{W}_B}$.
3.
$C$ is decomposed with the $W$ matrix initialized as ${{W}_D}$ and not updated obtain ${{H}_C}$.
4. The heldout matrix $A$ is then approximated by ${{W}_B}{{H}_C}$, which we will call $A^{\prime}$.

This is carried out with each block being used as $A$ and is repeated $n$ times with rows and columns of $X$ randomly shuffled, by default $n = 100$. The implementation of NMF in scikit-learn is used in cvaNMF [30].

Cosine similarity and ${{R}^2}$ are used as the two primary measures of correspondence between $A$ and $A\text{'}$. We calculate $CS( {A,A^{\prime}} ),\ $ the cosine similarity between flattened vectors of matrices $A$ and $A\text{'}$


\begin{eqnarray*}
CS\left( {A,A^{\prime}} \right) = \frac{{A \cdot A^{\prime}}}{{\|A\|\|A^{\prime}\|}}
\end{eqnarray*}

${{R}^2}$ is calculated as


\begin{eqnarray*}
{{R}^2} = 1 - \frac{{\sum {{{\left( {{{A}_{ij}} - A{{^{\prime}}_{ij}}} \right)}}^2}}}{{\sum {{{\left( {{{A}_{ij}} - \overline {A{{^{\prime}}_{ij}}} } \right)}}^2}}}
\end{eqnarray*}

We use median values for automated rank selection, and plot the distribution of values as box plots. This is implemented in cvaNMF as function ‘rank_selection’.

### Signature similarity

From different initial states NMF will converge on different solutions, meaning different signatures. After having selected the best solutions from among those from multiple starting states, we compare how similar each signature in the best solution is to it’s matched counterpart in all other solutions. Our expectation is that at a suitable rank (number of signatures), they will be highly similar across initializations, and lower at an unsuitable rank. A diagram of the process is given in Supplementary Fig. S18 along with pseudocode in Algorithm 1, and we implemented it as function ‘signature_similarity’ in cvaNMF.

From $n$ decompositions of the same input matrix $X$ with random initialization, we compare the signatures of the ‘best’ decomposition to the $n - 1$ other decompositions. The best decomposition is that which minimizes some criteria, by default we use reconstruction error. For best decomposition, and each of the other in $n - 1$, signatures may be similar but not in the same order in the signature matrices. We match signatures using the Hungarian algorithm [31] with cosine between signatures as the cost and seek to maximize cost. This provides a pairing of signature in the best decomposition to the other and a cost (cosine similarity) for the pairing. The cosine between matched signatures is the signature similarity, and we use as the signature similarity score for rank selection the mean value of signature similarity for all signatures and all $n - 1$ decompositions. The per signature distribution of signature similarity can also be plotted.


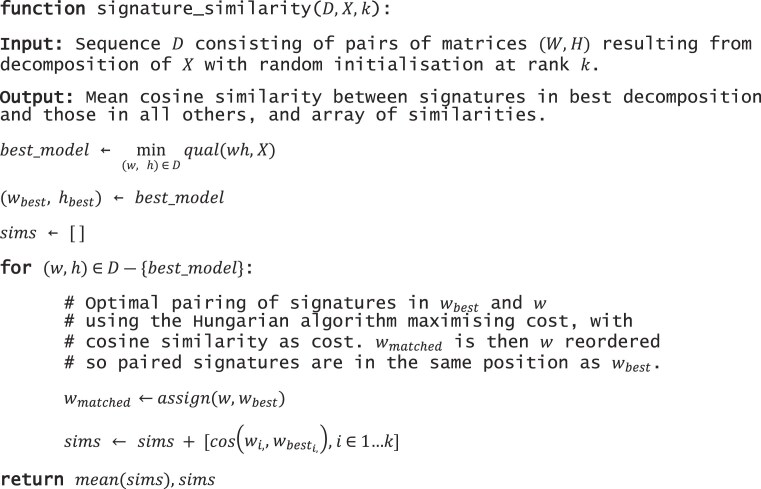

Algorithm 1: Pseudocode for signature similarity algorithm. From sequence D of decompositions with random initialization at rank *k*, we calculate cosine between signature vectors in the decomposition which minimizes a quality function (by default reconstruction error) and paired signatures in all other decompositions in *D*. The mean value is used in rank selection.

### Rank selection heuristics

For bicrossvalidation we select as the suggested rank the elbow point in the curve of cosine similarity and ${{R}^2}$. This is identified using the method given in Satopaa *et al.* [32] to identify the point of maximum curvature, as implemented in python package kneed [33]. This is implemented in the ‘suggest_rank’ function in cvaNMF, and by default uses the ‘online’ version of this method, which does not terminate at the first candidate. The parameters can be provided.

The stability-based methods typically do not show an elbow at the correct rank, so instead we use a custom heuristic to select the suggested rank based on local maxima to identify candidates and length of monotonic decrease to select among candidates: We use the following heuristic to select a rank from cophenetic correlation, dispersion, or signature similarity values. Let $v( k )$ be the value at rank $k$. First identify all local maxima as candidates $C$, considering only $v( {k - 1} )$ and $v( {k + 1} )$. Retain only those candidates $C$ for which $v( k ) \ge ( {1 - t} )max( {v( C )} )$. If there are multiple candidates remaining, select the candidate for which the value monotonically declines longest following this candidate rank. For our experiment here we use $t = 0.02$. This procedure is implemented in the ‘denovo’ module of the cvaNMF as function ‘suggest_rank_stability,’ and pseudocode given in Algorithm 2.


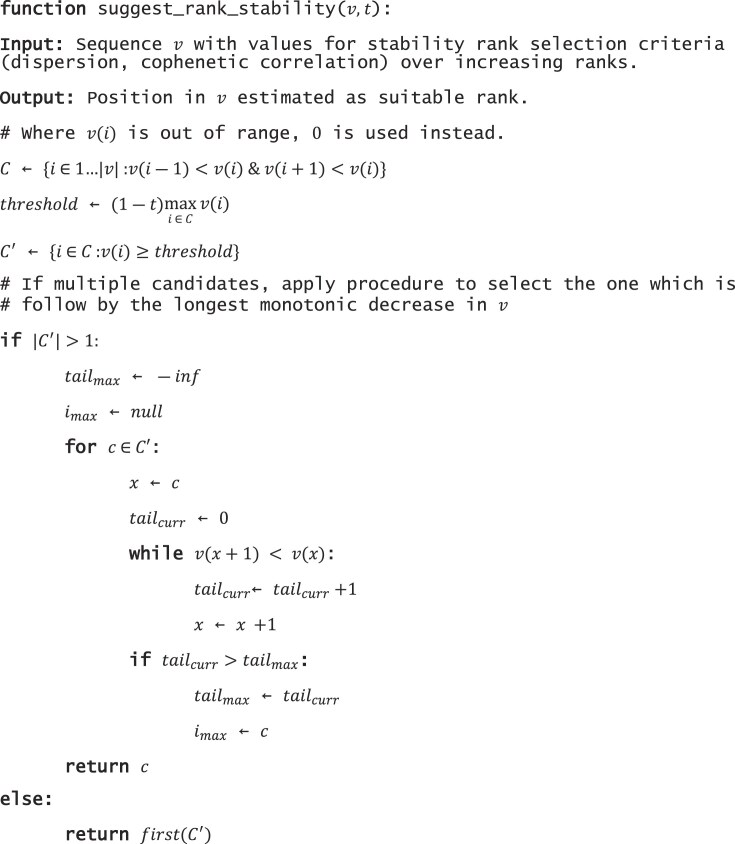

Algorithm 2: Heuristic for suggesting a suitable rank based on stability (cophenetic correlation, dispersion, and signature similarity) coefficients.

### Synthetic data generation

We adopt a method similar to the procedure used by Maisog *et al.* [34]. To create data with $k$ signatures, a $W$ matrix with dimensions $nk \times k$ is created. For signature $i \in 1 \ldots k$, rows $( {i - 1} )n + 1$ to $in$ are considered as features which are part of this signature, and given values drawn from a lognormal distribution with $\mu = 0.0,\ \sigma = 1.0$ in column $i$. To create signatures which are not orthogonal, as might be expected from real world situations such as biological systems, we additionally for each signature randomly select $p( {kn - n} )$ features initially assigned to other signatures and give them a weight in column $i$, from the same lognormal distribution. For our experiments here, we use $n = 20$ and $p = 0.25$ for $k\in 2..12$.

An H matrix with dimensions $k \times kn$ and filled with values drawn from a uniform distribution with range $[ {0,1} ]$. A sparsity of $s$ is then enforced by setting $skn$ entries randomly selected without replacement to 0. Here, we used $s = 0.25$.

Normally distributed noise is then applied to $WH$ from a distribution with $\mu = 0.0,\ {{\sigma }^2} = 0.25$. Any entries <0 are set to 0. This procedure is implemented in the ‘data’ module of the package as ‘synthetic_dense’. An example matrix is shown in Supplementary Fig. S1.

### Data decomposition and analysis

Rank selection time benchmarks were performed using a single virtual machine allocated 8 AMD EPYC 7601 cores at 2.2 GHz, 16 Gb of memory, running Ubuntu 22.04.5 LTS, and cvaNMF v0.5.0. A single random matrix was produced using the ‘synthetic_dense’ function in cvaNMF, with a scale similar to a genus level table from a metagenomic experiment (400 features and 1500 columns) and 5 signatures and h_sparsity set to 0.2. Rank selection was run 10 times, with 100 random initializations (or shuffles for bicrossvalidation) for ranks 2 to 15 inclusive, and default parameters for decomposition. Cophenetic correlation, dispersion, and signature similarity were all calculated from the same set of decompositions in each run.

The genus level abundance table used to generate Enterotypes from Keller *et al.* [9] was used (https://github.com/grp-bork/enterotypes), from which we removed the row representing unknown taxa (‘?’) before total-sum-scaling. For *Melilotus officinalis* soil and root microbiome, the ASV count table from Zhou *et al.* was summed to genus level and subsequently total-sum-scaled [35]. The class level fungal and bacterial count table from Bahram *et al.* [36] was used, from which we removed the row representing unknown taxa before total-sum-scaling. The cell-type annotations from the NSCLC cell metadata were used and converted to a cell-type abundance able, which was total-sum-scaled [37].

For all decompositions, rank selection used the parameters num_shuffles = 100, seed = 4928, init = random, beta_loss = kullback-leibler, and ranks = 2..20. Decompositions of the full matrix used the same parameters, with random_starts = 100. Rank selection and decomposition was performed using the cvaNMF nextflow pipeline for the Enterotype and global fungal and bacterial data. The NSCLC and *M. officianalis* data were smaller and thus processed on a local computer machine using the Jupyter notebooks included with cvaNMF.

Analysis of human gut metagenomes with disease signal: Representative signatures are defined as the smallest set of signatures required to represent 90% or more of the signature abundance in a sample as in Frioux *et al.* [17]. Single signature dominance (SSD) is when a sample has one representative signature (at >90% of total signature weight). Changes in number of representative signatures were performed using the Kolmogorov–Smirnov tests. We define the primary signature from a sample as that signature with the highest weight.

Changes in mean signature relative abundance in disease conditions were performed using linear models with disease and study as fixed effect for diseases without longitudinal samples, or linear mixed models with random intercepts for subject for those within longitudinal samples, using R package ‘lmertest’. We selected only studies which include both disease and control samples. Differences in estimated means were produced using R package ‘emmeans’, and *P*-values adjusted using BH adjustment.

For random forest models predicting disease status, the input matrix consisted of signature weights normalized by sum, and model fit for each sample. To avoid auto-correlation, we removed time series data, only retaining the first timepoint per individual for longitudinal studies. We selected only studies that contained at least 20 disease and 20 control cases.

Models were trained using data from one study, as the data are more likely to be matched in demographic and batch effects, and a separate model created for each disease in the study. We trained random forest models for binary classification using the ranger package in R, measuring importance using Gini impurity score, as well as growing classification forests and probability forests. Models were evaluated using both leave-one-out cross-validation (LOOCV) and stratified five-fold cross-validation (FFCV) (Supplementary Fig. S9) to retain class balance. Performance was measured by ROC-AUC, MCC, and F1 score, calculated using the measures package. AUC from both Leave-One-Out and 5-fold crossvalidations were similar (±0.052, Supplementary Table S1).

In global diversity data we excluded composting and hot springs from aerobic versus anaerobic analysis as environments of these types could fall in both categories, and it was unknown for each sample. LMM were fitted with the formula ‘aerobic + host + aerobic*host + (1|Country)’ using R packages lmer and analysis using emmeans.

Geographic distance between *M. officianialis* sampling sites was calculated using the distance.distance function from ‘geopy’ based on latitude and longitude provided in supplementary material of Zhou *et al.*

## Results

### Performance of novel and established measures for optimal signature number

**Figure 1.**
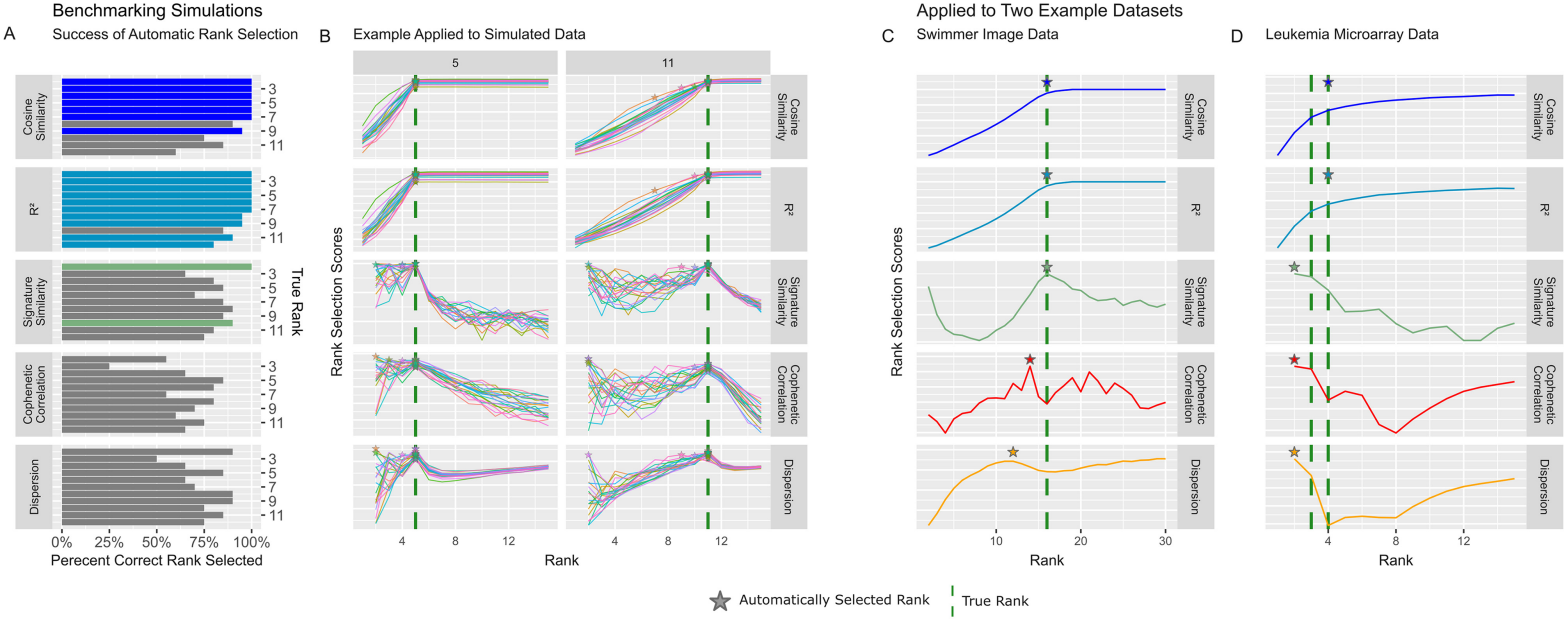
Performance of rank selection methods identifying rank in simulated data with a known rank and example data. (**A**) Performance of rank selection criteria on simulated benchmark data. For each rank from 2 to 12, 20 datasets were generated, % of (automatically) correctly identified ranks of the 20 test cases per rank are shown. Bars are only coloured if the method performed best at this rank. (**B**) Rank selection on simulated data with true ranks 5 and 11. Bicrossvalidation plots the median for all folds. Each simulated dataset (of *N* = 20 total) is visualized by lines of different colours in subfigures. (**C**) Rank selection for Swimmer image data. Line for bicrossvalidation methods indicates median of values across random initializations. (**D**) Rank selection for microarray RNA data of leukaemia. Bicrossvalidation methods are in blues, and stability methods in red and orange, signature similarity in green. Stars are placed at the rank that was suggested automatically by cvaNMF, the dashed green line indicates the true rank for each respective dataset.

The number of signatures in a decomposition is described as the rank; selecting a suitable rank for a given dataset is central to obtaining a meaningful decomposition of data into signatures. Therefore, we compared the performance of rank selection methods on synthetic and real data where the number of ranks was known *a priori*. Five methods were compared, including two measures based on block bicrossvalidation [28, 29], two stability based methods (cophenetic correlation, dispersion coefficient) [20, 27], and a novel method we named ‘signature similarity’ (Fig. 1A), that are implemented in cvaNMF.

In a synthetic data benchmark, we simulated compositions of rank 2 to 12. Features were shared across signatures and multiple signatures were allowed to represent the simulated samples (Materials and methods, Supplementary Fig. S1). In addition, two published datasets previously used in benchmarking tasks were benchmarked: the swimmer dataset which is image data with a simple stick figure representation of a swimmer in 16 possible limb positions, and RNA expression microarray data from leukaemia samples, which has been previously found to contain either 3 or 4 signatures (Methods) [20, 38].

Bicrossvalidation generates crossvalidated estimates of the true dataset rank, that are evaluated using either cosine or ${{R}^2}$ similarity between hold-out and training data. In our simulations, this is superior in determining the true rank of the synthetic data, identifying the correct rank in 86.4% and 87.7% of cases for cosine similarity and ${{R}^2}$, respectively (Fig. 1A), making bicrossvalidation the best performing method tested. However, at ranks >10 an elbow point is identified too early by automatic algorithms, that is in most cases evident from visual inspections, highlighting the usefulness of visualizations provided by cvaNMF (Fig. 1B). This is similar to previous benchmarks that showed degrading performance for bicrossvalidation at higher ranks [34]. Therefore, visual inspection is in general recommended beyond the automatic cvaNMF rank recommendation.

Our novel ‘signature similarity’ method provides a quantification of how often the same features are selected in a signature between multiple random initializations. This can assist interpretation, by identifying less reliable signatures, or to identify the rank that has the most robust signatures. This approach performed less well (82% correct rank) than the computationally more complex bicrossvalidation, but outperformed both stability-based rank determination methods, cophenetic correlation (60.5%) and dispersion coefficients (68.2%). Our implementation of signature similarity is two orders of magnitude quicker to calculate for generated decompositions than cophenetic correlation or dispersion. On benchmark data, after multiple decompositions were generated for 14 different ranks, signature similarity was calculated on median in 7 s, compared to 5 m and 25 s and 5 m and 23 s for cophenetic correlation and dispersion, respectively (Supplementary Fig. S2). In the case of bicrossvalidation, this method is not based on assessing decompositions of the full matrix so cannot be directly compared, but the total execution time for bicrossvalidation for the same parameters extends to median 5 h 23 m and 38s.

In the swimmer dataset, bicrossvalidation and signature similarity performed well (Fig. 1C), correctly identifying the 16 signatures that represent the 16 limbs of stick figures. Stability based methods underestimated rank, suggesting 12 for dispersion and 14 for cophenetic correlation, with dispersion having a local minima at the correct rank. At the bicrossvalidation and signature similarity suggested rank of 16, each signature represents one limb, while at ranks 12 and 14, as suggested by dispersion and cophenetic correlation, multiple limbs are combined in signatures (Supplementary Fig. S3). Similarly, in the leukaemia microarray data, bicrossvalidation suggests a rank of 4; both ranks 3 and 4 have been identified as suitable in the literature [20]. Both stability-based measures and signature similarity suggest rank 2 (Fig. 1D), therefore underestimating the manually identified rank.

Overall, bicrossvalidation performed the best at selecting the true rank of synthetic and real datasets. The newly introduced ‘signature similarity’ outperformed cophenetic correlation and dispersion rank measures, with signature similarity having a lower computational cost.

### Gut microbiome ‘enterosignatures’ are robustly recovered across different datasets and approaches

cvaNMF was first used to re-identify enterosignatures in a large gut metagenome dataset (*N* = 16 193) that was recently published by Keller *et al.* [9]. Here the authors reported finding three enterotype (ET) clusters identified via fuzzy clustering of genus level abundances, typified by *Firmicutes, Bacteroides*/*Phocaeicola*, and *Prevotella* [9].

**Figure 2.**
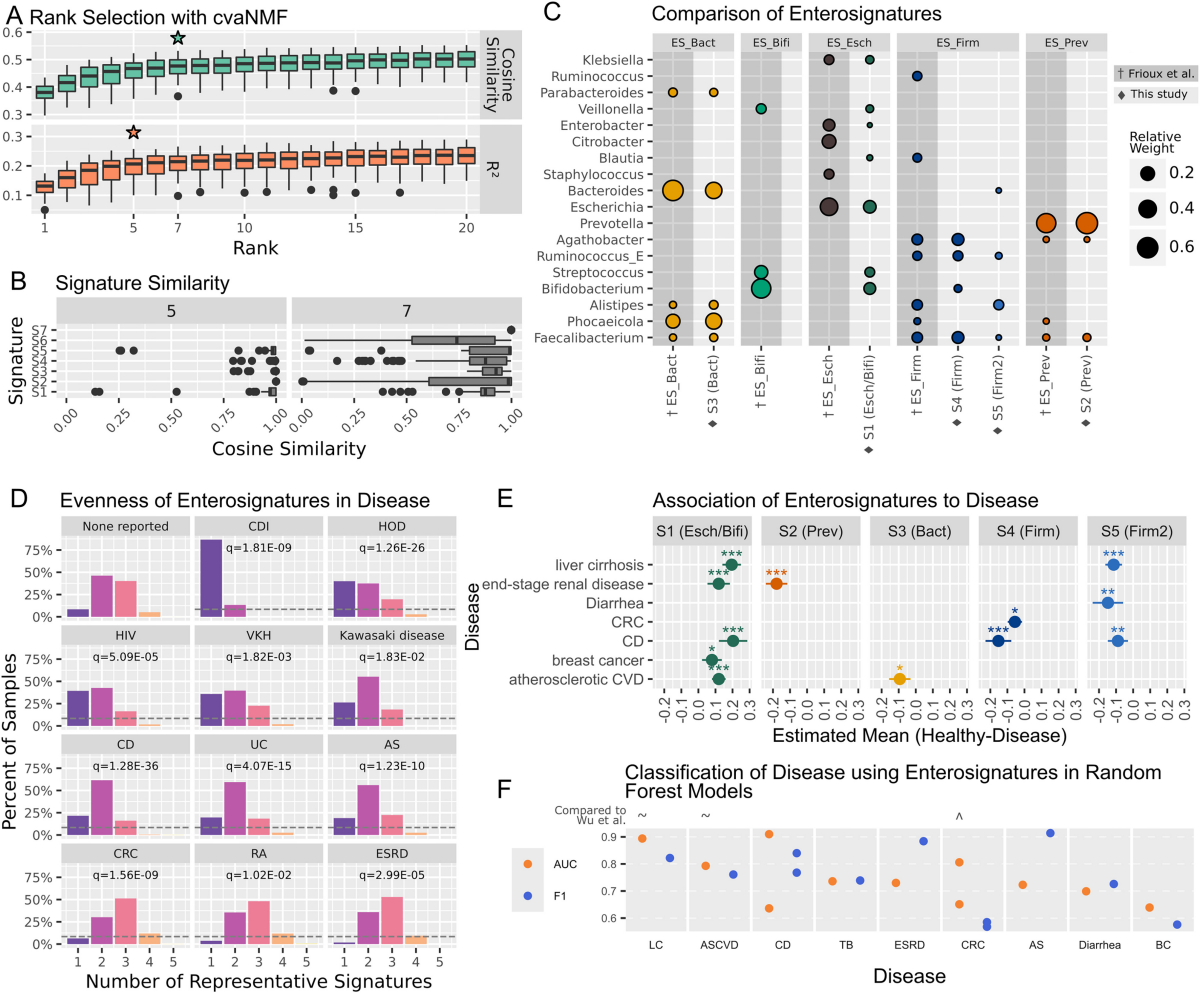
Signatures based on gut microbiome data from Keller *et al.* [9], and their association to disease conditions. (**A**) Bicrossvalidation based rank selection for the Keller *et al.* genus level table. Stars indicate the heuristically suggested values for suitable rank. (**B**) Signature similarity for models with 5 and 7 signatures, showing how similar signatures from random initializations were to those in the best decomposition. (**C**) Comparison of signatures recovered in Frioux *et al.* [17], and in this paper calculated *de novo* from Keller *et al.* data. Only genera which make up 1% of the total weight are shown. Signatures from Frioux *et al.* have a dark background, and signatures from Keller *et al.* are grouped with their closest matching signature. (**D**) Number of representative signatures in diseases which differ significantly from samples with no disease reported (Kolmogorov–Smirnov test, BH adjustment, *q* ≤ 0.05). Dashed line indicates the level of SSD in non-diseased samples. (**E**) Differences in mean signature values between healthy and diseased samples from the same study. Positive values indicate the mean is higher in diseased samples. Significance indicated by *q* < 0.001 ***, *q* < 0.01 **, *q* < 0.05 *. (**F**) Prediction of disease state via 5-fold crossvalidated random forest models for different diseases. Each point represents one study. Only those models achieving > 0.625 ROC-AUC are shown. Top row of labels shows comparison of ROC–AUC for our best performing model for a disease to the best performing model in Wu *et al.* [15] for the same disease. ‘^’ indicates our model performed better, ‘∼’ equivalent performance (±0.01), and ‘v’ that our model performed worse. Disease abbreviations: CDI = Clostridioides difficile infection, HOD = hemato-oncological disease, VKH = Vogt–Koyanagi–Harada disease, CD = Crohn’s disease, UC = ulcerative colitis, AS = ankylosing spondylitis, CRC = colorectal cancer, RA = rheumatoid arthritis, ESRD = end-stage renal disease, LC = liver cirrhosis, TB = tuberculosis, ASCVD = atherosclerotic cardiovascular disease, BC = breast cancer.

Using cvaNMF’s bicrossvalidation rank selection on the same genus level data, we identified either five (based on *R*^2^) or seven (based on cosine similarity) signatures (Fig. 2A) [9]. We chose five signatures, because Signature similarity suggested a more robust feature representation at this rank (Fig. 2B). Each signature has been numbered S1 to S5, and given a name based on the taxonomic groups for which it has highest weights: e.g. S3 (Bact) has high weight for Bacteroides.

Overall the *de novo* recovered signatures are both highly similar to enterotypes and enterosignatures: S3 (Bact) and S2 (Prev) were also described in the enterosignatures from Frioux *et al.* [17], and to the enterotypes of Keller *et al.* S3 (Bact) has high weight for *Bacteroides* and *Phocaeicola*, and S2 (Prev) high weight for *Prevotella* (Fig. 2C). S1 (Esch/Bifi) has high weights for *Escherichia* and *Bifidobacterium*, taxa which did not form an enterotype when clustered by Keller *et al.* but were previously detected as two separate enterosignatures: ES_Esch and ES_Bifi that are mostly found in infants. Infant samples were excluded from the Keller *et al.* dataset used here, but the *Bifidobacterium/Escherichia* signature could still be recovered from adult gut metagenomes, highlighting the importance of accounting for non-dominant signatures. However, S1 (Esch/Bifi) appears combined with other potentially harmful bacteria such as *Streptococcus* and *Enterococcus*, making it a potentially pathobiont signature, getting refined into separate signatures at ranks >6 (Supplementary Fig. S4). Finally, two signatures, S4 (Firm) and S5 (Firm2), represented the *Firmicutes* bacterial guilds.

Both sets of signatures are very similar (Supplementary Fig. S6): Bacteroides and Prevotella signatures show 0.99 and 0.91 cosine similarity, respectively, and <0.1 to any others. S1 (Esch/Bifi) learnt from the Keller *et al.* data show 0.82 cosine similarity to the sum of ES_Esch + ES_Bifi from Frioux *et al.*; similarly ES_Firm from Frioux *et al.* shows 0.81 to the sum of Firmicutes signatures S4 (Firm)+ S5 (Firm2) learnt from Keller *et al.* This similarity is noteworthy, as the taxonomic profiling in Frioux *et al.* and Keller *et al.* was conducted with two completely different approaches: Frioux *et al.* using an assembly based approach (MG-TK [39] and GTDB r207), while Keller *et al.* profiled taxa used an reference-based approach (mOTUs3 [40] and GTDB r202), demonstrating robustness of signature-decomposition to methodological differences.

When discretizing our gut signatures by taking the primary signature (the signature with highest weight), we found cluster assignments to be highly consistent (83% sample match) with the Enterotypes (ET) by Keller *et al.* (Supplementary Fig. S5). The adjusted Rand Index between the ES and ET clustering’s was 0.47 (*P*< 0.001, permutation test [41]), suggesting significantly similar structures represented. The majority of mismatched labels were samples assigned to S1 (Esch/Bifi, 9% of all samples), which has no equivalent ET. This illustrates that signatures can capture the same broad structures as clustering methods, while identifying the contribution of additional biologically relevant but low abundance groups of taxa such as Escherichia and Bifidobactrium. Further, we also found concordance between the methods in a significant correlation between model fit, which describes how well the model reconstructs the input data for each sample, and the dysbiosis score of Keller *et al.* (*r* = −0.44, *P*< 0.001, Supplementary Fig. S7).

### Enterosignatures can predict diseases with similar accuracy to genus and species level models

Metagenomes from healthy subjects were predominantly a mix of three (46%) or two (40%) signatures. SSD, where one signature contributes at least 90% of total weight for the sample, was the least often observed (8%, Fig. 2D). Several diseases showed a different distribution in the number of representative signatures (Kolmogorov–Smirnov test, BH adjustment, *q* ≤ 0.05) frequently displaying an increase in rate of SSD compared to healthy samples. Healthy gut microbiomes were most often a mixture of signatures, and dominance by one was associated with many of the diseases included in the data [all except colorectal cancer (CRC), rheumatoid arthritis, and renal disease].

Disease state could in many cases also be ascribed to a shift in signature composition (Fig. 2E). Significantly increased S1 (Esch/Bifi) was the most frequent change, occurring in liver cirrhosis, end-stage renal disease, Crohn’s disease (CD), breast cancer, and atherosclerotic cardiovascular disease (ASCVD). A decrease in Firmicutes signatures S4 and S5 was observed in liver cirrhosis, diarrhea, CRC, and CD. Decreases to S2 (Prev) and S3 (Bact) occurred in renal disease and ASCVD, respectively. In ulcerative colitis (UC), we observed an increase in SSD of S3 (Bact) (Supplementary Fig. S8). Samples for some diseases originate from multiple studies recruited from different populations, and we account for the between-study differences by including it as a fixed effect in our statistical models. However, other potentially confounding factors such as age or diet were not consistently available across different studies and could therefore not be accounted for.

We tested if signature weights can be used in isolation to classify diseases via random forest (Fig. 2F). Indeed, 12 out of 27 tested diseases could be identified with an AUC–ROC > 0.625. CD was most reliably identified (best AUC–ROC = 0.91, F1 = 0.84), with the ES-based model performing slightly better than our results from 16S amplicon sequencing data [42]. Congruent to our univariate testing (Fig. 2E), all diseases with significant changes had a ROC–AUC of > 0.625. Tuberculosis (TB) and ankylosing spondylitis (AS), which were not significantly different in linear model analysis, were moderately successfully classified (ROC–AUC = 0.70. 0.67, and 0.64, respectively). Comparing these results to performance of models trained on the original genus level data, we found comparable F1-scores (±0.1) in 77% of all models when evaluated with 5-fold crossvalidation, with AUC within the same range in 44% of models (Supplementary Fig. S9).

A recent study by Wu *et al.* [15] trained random forest models on a set of health associated species identified by network analysis to form two competing communities. Six tested diseases overlapped with our analysis. The enterosignature model performed worse in three diseases, with our best performing models for inflammatory bowel disease (IBD), schizophrenia (SCZ), and type 2 diabetes (T2D) (Fig. 2F). However, for CRC, LC, and ASCVD, enterosignature based models had similar or even better performance.

Signatures identified by cvaNMF can therefore effectively describe gut microbiomes congruent with established research, while allowing the identification and classification of disease states at similar performance to state-of-the-art approaches.

### Fungal and bacterial signatures in host- and environment-associated microbiomes

We next investigated how well NMF signatures can capture a more diverse and heterogeneous set of microbiomes, relying on 13 495 metagenomes, representing 25 distinct biomes of both bacterial and fungal taxa [36]. In these data, we previously identified three groups of biomes using hierarchical clusters (HC), with HC1 containing environmental microbiomes, HC2 surface and skin microbiomes, and HC3 gut microbiomes from various animals.

**Figure 3.**
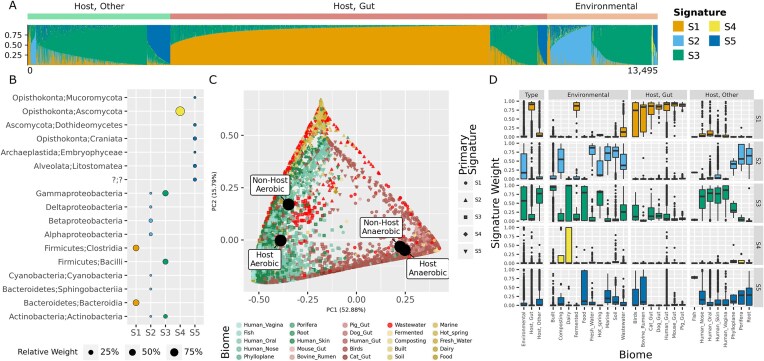
Class level taxonomic abundance of 13 495 samples from multiple biomes decomposed to five signatures. (**A**) Bottom is the relative abundance of each signature in samples. Coloured bar at the top indicates whether the sample is from a gut, other host associated, or environmental source. (**B**) Relative weight of classes in each signature, with only those >4% shown. (**C**) PCoA of signature weight matrix. Each point represents a sample, with shape showing the signature with highest weight in the sample, and colour the biome. Colours show gut, other host, and environmental samples. Centroids for host and non-host associated aerobic and anaerobic samples are shown with labelled points. (**D**) Distribution of signature relative abundance across biomes.

Using cvaNMF, we found a five-signature decomposition best describing both fungal and bacterial classes (Fig. 3A and B). Bicrossvalidation suggested a rank of 4 or 5 based on *R*^2^ or cosine similarity respectively. We selected a rank 5 decomposition as cosine similarity remains stable between 5 and 10 signatures, suggesting that adding signatures beyond 5 does not improve the model (Supplementary Fig. S10). We found that the abundance of these signatures corresponds to source of the sample (Fig. 3A): S1 is typically the most abundant signature in gut samples, while S2 is more abundant in environmental and some non-gut host associated microbiomes such as root and phylloplane samples (Fig. 3D). The first three signatures exclusively represent bacterial taxa (Fig. 3B), that are largely concordant with the prior HC (Supplementary Fig. S11) [36]. S1 may represent anaerobic environments, rather than strictly the gut, as it is also highly abundant in fermented samples (Fig. 3D). Using an ordination of signature weights, we visualized relations among biomes and showed samples clustering largely by the HC groups, apart from wastewater samples (red in Fig. 3C). Wastewater samples are likely to have inputs from a mixture of sources, and in the decomposition appear correspondingly as a mixture of the three bacterial signatures, rarely taking a high weight for a single signature (Fig. 3D). Biomes mixed from several sources, such as mixed wastewater, are typically hard to fit into a hierarchical clustering structure, while the NMF model can uncover intuitively this mixed nature, as well as the shared similarity with human skin, oral, and gut microbiomes.

An advantage of signatures compared to hierarchical clustering is interpretability, the feature weights allowed us to easily describe which bacteria are important in a given environment. For instance, bacterial signature S2 is prevalent in fresh water and marine environments, and includes the phylum Cyanobacteria which is important for primary production in marine aquatic ecosystems. Proteobacteria are also found among the important phyla in S2, of which many taxa play important roles in the nitrogen cycle in both aquatic and soil environments, the latter also being enriched for S2.

The included environments are heterogeneous and relationships between taxa may differ in each. These different roles by higher order taxa are nonetheless combined in our signature approach. For example Actinobacteria and Gammaproteobacteria appear in both non-gut bacterial signatures S2 and S3, but these only make a subset of all microbes representing S2 and S3. Given the distinct prevalence of signatures (Supplementary Fig. S12), even partially overlapping taxa between signatures do not invalidate that very different ecosystem conditions can be captured.

Two further signatures (S4 and S5) describe the distribution of eukaryotes, with S4 being almost exclusively Ascomycota, and S5 a mix of lower weights for a range of fungi and other eukaryotic taxa (Fig. 3C). *S*ignature S4 has a higher median weight in gut samples (*P*< 0.00001, Mann–Whitney *U*-test), as well as high weight in composting, dairy, porifera (sponge), and some soil samples, potentially representing environments where degradation of organic matter is important (e.g. leaf litter or animal proteins [43]). S5 has a wider distribution among most non-gut microbiomes, with very low weights in most gut samples, reflecting the orders of magnitude difference in bacterial:fungal cell ratios in the gut [44] and the relative importance of Ascomycota in the gut mycobiome [45].

There are few positive relationships between signatures, and those between the abundance of fungal and bacterial signatures across samples are weak (Supplementary Fig. S13). S2 (environmental microbiomes) correlates to S4 and S5 (fungal and eukaryote dominated) at respectively *r* = 0.19 and *r* = 0.26 (Spearman correlation, *q* ≤ 0.001), while S3 (skin and surface) correlated to S5 (*r* = 0.05, *q* ≤ 0.001), which contains fungi as well as other eukaryotic organisms. These low correlations between signatures, specifically S4 and S5, reflect an independence among fungal and bacterial compositions not described at scale before. S1 (gastrointestinal) negatively correlated with all other signatures, potentially reflecting the division between aerobic and anaerobic microbes, the latter being a stronger separation among microbiomes than the distinction host versus environmental. Indeed, for all bacterial signatures (S1–S3) the difference between estimated means of aerobic and anaerobic samples was significant for both host and non-host associated samples, while the reverse was n.s. (Supplementary Fig. S14).

Therefore, the uncovered signatures robustly describe the structure of global fungal and bacterial microbiomes from varied biomes, identifying similar communities, their mixtures in different sample types as well as dependencies among these.

### Root associated microbiome signatures stable across geographic distance

We next investigated the environmental microbiome in more detail, choosing a study that investigated soil and root microbiomes at different spatial scales. Bacteria are found in association with plant root structure, the rhizosphere, within plant tissues, the endosphere, and with the exposed plant surface, the phyllosphere [46]. Plant microbial community compositions vary not only with species and soil community but also with plant development stage and season. Zhou *et al.* identified separate communities between soil and root compartments for young and mature *M. officinalis* from 24 sites along a 60 km stretch of the Yellow River Delta in China [35]. They found young plants exerting less influence on the rhizosphere microbiome, indicated by a more similar composition of soil and young rhizosphere community composition, and that geographic distance between plants had a stronger influence on soil and mature plant rhizosphere, than in young plants.

**Figure 4.**
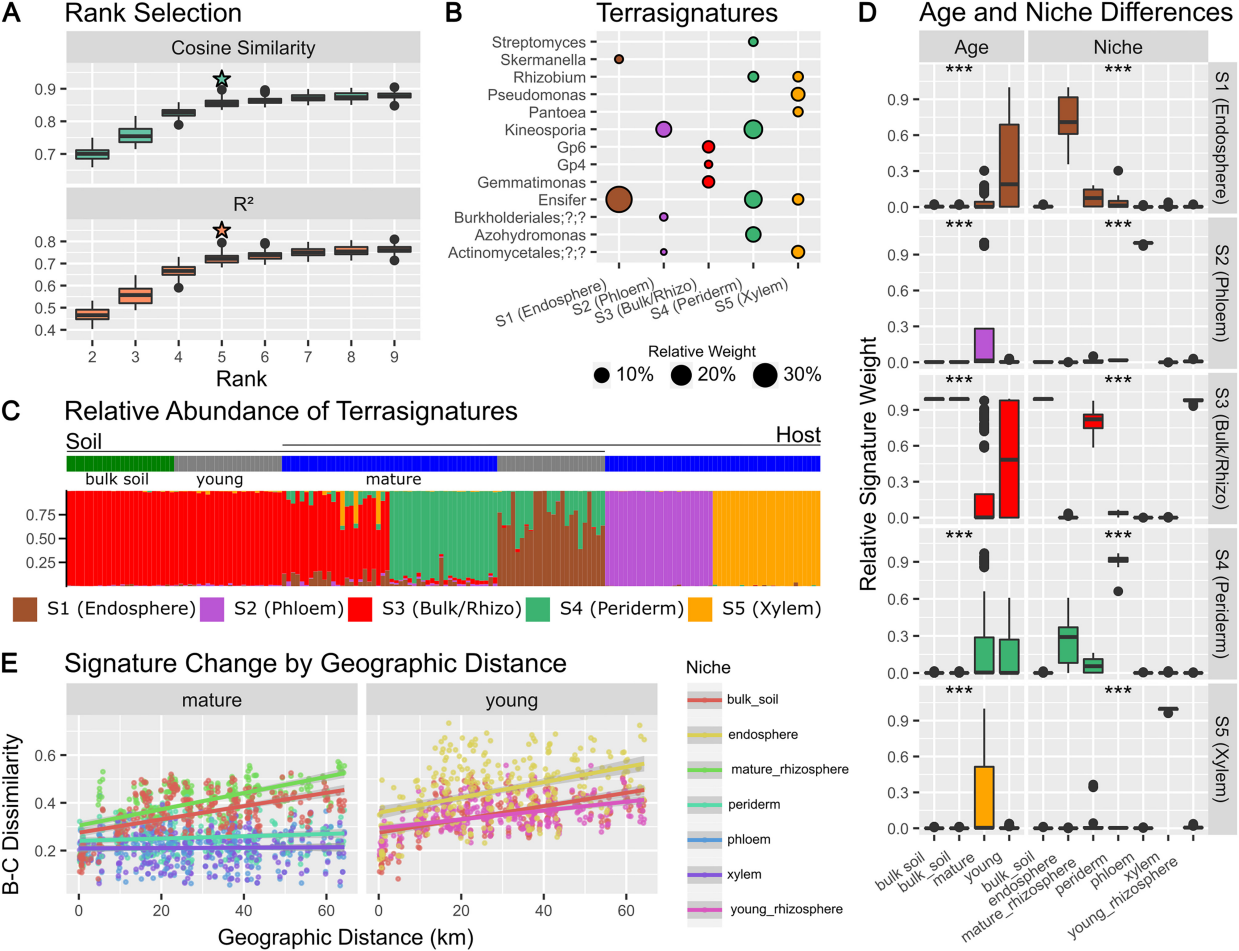
Decomposition of soil and root microbiome of young and mature *M. officinalis*. (**A**) Bicrossvalidation based rank selection, suggesting rank 5 as suitable. (**B**) Weight of genera in signatures, with only those over 4% shown. (**C**) Relative abundance of each signature in different plant niches and bulk soil. For young plants the endosphere encompasses all plant tissue compartments (periderm, phloem, and xylem) which are separated out in mature plants. (**D**) Distribution of signature relative abundance by plant age and sample niche. Significance tested using Kruskal–Wallis test with BH adjustment, indicated by *q* < 0.001 ***. (**E**) Relationship between signature dissimilarity (pairwise Bray-Curtis dissimilarity distance using signature weights) and geographic distance for each niche.

We applied cvaNMF to the genus composition of this dataset: five optimal signatures were identified, here termed ‘terrasignatures’ as the first of their kind (Fig. 4A). Three of these signatures could be associated with specific environments: S2 (Phloem), S4 (Periderm), and S5 (Xylem) (Fig. 4C and D). The soil microbiome was represented by S3 (Bulk/Rhizo), with the typical high diversity of the soil microbiome being reflected by only a few genera having high weight, with a long tail of low weight genera (Supplementary Fig. S15). This signature is not restricted to only soil, it is also the primary signature in the rhizosphere of mature and young plants (Fig. 4C and D). Corroborating the conclusions from the original publication, the rhizosphere in young plants is almost entirely composed of S3 (Bulk/Rhizo), while in mature plants signatures which could originate from within the plant have a stronger influence.

Young plants do not yet have experimentally separable periderm, phloem, and xylem tissues, their internal tissue is therefore described as ‘endosphere’. In mature plants, distinct terrasignatures clearly associate with periderm (S4), phloem (S2), and xylem tissues (S5). In contrast, we found the endosphere of young plants to be a mixture of S4 (Periderm) and S1 (Endosphere), dominated by the nitrogen fixing genus *Ensifer* (Fig. 4B), representing the symbiotic rhizobia *Ensifer meliloti*. This genus was also present in the mature periderm signature S4 (Periderm) with lower weight, with a mixture of spore forming bacteria *Kineosporia* and *Streptomyces*, and other nitrogen fixing bacteria such as *Rhizobium* and free-living *Azohydromonas* being an important part of this signature with a symbiotic role beneficial to the plant.

Zhou *et al.* further observed that the geographic distance between sites was positively associated with the dissimilarity between bacterial communities. We tested if the five terrasignatures offered enough resolution to detect such a sensitive gradient signal; indeed, the mature rhizosphere, young plant endosphere, and bulk soil correlated to geographic distance (Fig. 4E). Internal microbiome signature abundances had no correlation to geographic distance, suggesting that mature plants select for a different rhizobiome but not internal microbiome dependent on geographic location, while for young plants this selection may take place at the periderm or internally.

The plant samples are separated by up to 65 km, and Zhou *et al.* note a high degree of variation in soil conditions between soil sites [35], which may drive these associations rather than distance alone. Soil conditions are correlated to geographic distance (Mantel test *r* = 0.29, *P*= 0.002), and when accounting for geographic distance we only saw significant correlations between soil and signature distance in the rhizosphere (*r* = 0.14, *P*= 0.012, partial Mantel test), concordant with results from Zhou *et al.* [35].

Thus, our signature-based approach successfully identified the bacterial guilds that describe most plant-associated microbial niches, from bulk soil to internal plant compartments. Because we can describe each sample as a mixture of signatures, more subtle differences such as distance-based microbiome-similarity decay or mixture of communities in some compartments could be described.

### Cell-type signatures associated with disease progression can be identified non-small cell lung cancer single-cell sequencing data

To demonstrate the wider applicability of cvaNMF, we last investigated a mixture of different cell types, relying on single cell sequencing data from the non-small cell lung cancer (NSCLC) cell atlas [37], combining 556 samples from tumors and healthy tissue from patients with NSCLC, including adenocarcinoma and squamous cell carcinoma, as well as from some non-cancer controls. Specifically, we used the relative abundance of 33 different cell types in each sample as input, using cell labels identified by Salcher *et al.* [37]

**Figure 5.**
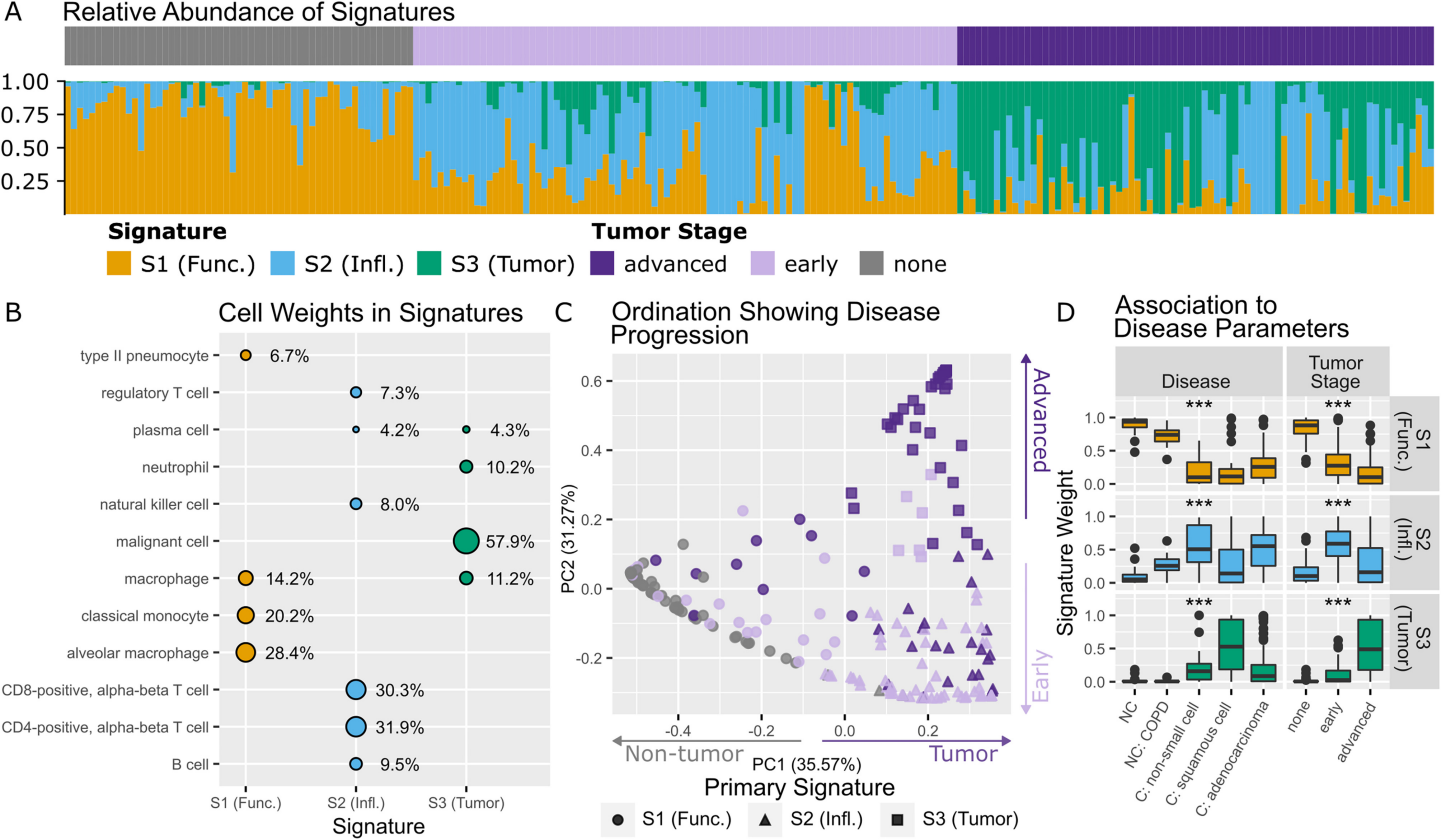
Analysis of cell type composition data of lung tissue, from participants with carcinomas, with chronic obstructive pulmonary disease or non-cancerous tissue. (**A**) The relative weight of cell types in samples. The top bar indicates tumor stage, the bottom the relative abundance of each signature in a sample. (**B**) Weight of cell types in each signature, with only those above 4% shown. The cells with high weight were predominantly immune cells, other cell types had weight below 4% toal. (**C**) Principal coordinates analysis on the relative signature weight matrix, using Bray–Curtis dissimilarity. Colour shows the tumor stage, while shape shows the primary signature. (**D**) Signature relative weight and model fit across disease types and tumour stage. *** indicates a BH adjusted *P*-value ≤ 0.01 from a Kruskal–Wallis test including all categories in disease and tumour stage. C denotes samples from tumour, NC from non-tumour tissue. ‘C: non-small cell’ are NSCLC samples for which a specific type label was not available.

Rank selection using bicrossvalidation suggested three signatures, with all three signatures having a high median stability (Supplementary Fig. S16). In this decomposition, S1 (Func.) appears to represent typical lung function (Fig. 5B). It has high weights for type II pneumocytes involved in gas exchanges, along with immune cells involved in removal of microorganisms such as monocytes and macrophages, and particulate matter by alveolar macrophages. S2 (Infl.) represents immune function targeted towards host cells, with T-, B-, and NK-cells having high weights. Finally, over half of the signature weight of S3 (Tumour) is malignant cells, that represent cancerous cells, along with weights for macrophages, shared with non-cancerous S1 (Funct.). Immune cells form part of the tumour microenvironment, and macrophages being present in both signatures may be due to the differentiation between M1 and M2 macrophages, which show anti- or pro-tumour effects [47, 48], subtypes which are not identified in the NSCLC atlas.

Disease development shows a progression through these signatures, with healthy tissue being mostly S1 (Func.), S2 (Infl.) increasing in early tumour stages, and cancerous signature S3 (Tumour) increasing in advanced tumours (Fig. 5D). A PCoA on the signature weights demonstrated that the decomposition can separate tumour from non-tumour samples (axis 1), as well as identifying tumour stages (axis 2, Fig. 5C).

Differences are significant between tumour stages for all signatures (Fig. 5D). Post-hoc tests (Dunn’s test, BH multiple testing adjustment) show significant differences among tumour types as well as in comparison to non-cancerous samples. Adenocarcinoma and non-small cell cancers are significantly different (*q* < 0.01) to squamous in having a higher relative abundance of inflammatory immune cell signature S2 (Infl.). S3 (Tumour) is significantly different (*q* < 0.001) between adenocarcinoma and squamous cell carcinoma, with a higher relative abundance in squamous cell carcinoma indicating that this tumour has a higher proportion of malignant cells and macrophages.

Our NMF model has the potential to describe additional conditions, as a small number of non-cancerous samples were from subjects with chronic obstructive pulmonary disorder (COPD), an inflammatory disease caused by exposure to a range of factors, such a tobacco smoke. Samples from these individuals showed a significantly altered signature composition compared to other controls (Dunn’s test, *q* < 0.05), with a raised relative abundance of the immune-cell signature S2 (Infl.), whose cells such as CD4+, CD8 + T-cells are involved in producing the inflammatory immune response in COPD (Fig. 5D).

## Discussion

Initially introduced in the context of computer vision, NMF has been applied to biological problems, including identifying groups of genes associated with leukaemia subtypes [20], and identifying mutational signatures in cancer [21]. Since then, it has emerged as a broadly applicable machine learning approach for dimensionality reduction to decipher biological data. The non-negativity constraint provides and intuitive and interpretable description of data, allowing a straightforward understanding of both samples (as a mixture of signatures), as well as the contribution of original features (e.g. taxa) to each signature. Dissimilarity based dimension reduction techniques such as PCoA are widely used in ecology to identify similar broad structures but require additional steps to relate to original features. Other approaches common in topic modelling such as latent Dirichlet allocation (LDA) have been applied to biological data. LDA has a very similar set of assumptions as NMF describing samples as non-negative mixtures of topics that are themselves probability vectors over the observed words or taxa in our case. In fact, LDA can be viewed as a probabilistic extension of NMF with an explicit multinomial sampling allowing application to discrete data. Comparisons in document analysis find LDA to produce more signatures which are similar and widely shared across documents, while NMF signatures tend to be more distinct [49], and selecting between these methods may depend on which of these properties is more desirable and whether data is continuous or discrete. Several decomposition methods have been utilized, such as independent component analysis (ICA) [50–52] or latent process decomposition (LPD) [53–55]. They share some of the useful properties of NMF, i.e. identifying a small set of latent features whose mixture can describe a sample. ICA is used frequently in data where directionality has biological meaning, such as gene expression, or in measurements of brain activity [56]. However, when studying abundance data which is strictly non-negative, NMF has the distinct benefit of providing signatures with intuitive biological interpretation, making it a valuable tool for intuitively interpreting structures in high-dimensional data when seeking to identify structure in novel data. Such unsupervised data exploration is often held back by selecting a suitable rank (number of signatures/clusters) to meaningfully interpret the data. With cvaNMF we provide an easy-to-use interface to powerful algorithms for selecting optimal ranks, that also can take advantage of high-performance computing (HPC) infrastructures to interrogate datasets of any size.

In the pipeline, we provide a domain independent, memory optimized implementation of bicrossvalidation. While some of these approaches exist in other software, these are either domain specific (SigProfiler [21, 57], Ecotyper [22]), do not include rank determination (PyCoGaps [58]), or provide only clustering based rank determination methods (nimfa [59]). Additionally, cvaNMF provides many visualization and analysis methods, including plots for rank selection, feature weight, relative signature weight, PCoA ordination, and univariate testing to further guide rank selection and analysis. Our benchmarks show that bicrossvalidation performs better than the commonly used dispersion and cophenetic correlation coefficients in both synthetic and experimental data, although having a higher computational cost. In contrast, our newly developed ‘signature stability’ measure is faster to calculate than any other measure, and reaches a higher precision than dispersion and cophenetic correlation, as well as providing additional insights into signatures (Fig. 2D).

Our simulated benchmarks assume a single optimal rank. Real data may have an underlying structure which is well described at multiple ranks, rather than a single optimal rank [60]. For instance a bacterial guild may contain a subset of taxa which show competitive exclusion; a low rank decomposition could suitably describe the guild overall, with increasing ranks capturing the competitive subgroups. Postprocessing methods have been applied to LDA models [61, 62], identifying signatures which are coherent across ranks, and those which show refinement with increasing rank, to allow investigating data at multiple levels of resolution. Plots provided by cvaNMF of signature similarity at multiple ranks (e.g Fig. 2B) can assist in identifying consistently detected signatures at a given rank and which vary across initializations.

The power of cvaNMF was demonstrated by analysing a set of key biological datasets. First, we revisited the clustering of gut microbiomes, exemplified by Enterotypes [11], that have been investigated using a number of methods over the years [14], including recently ‘Enterosignatures’ [17]. Here, we reanalysed a large dataset with 16 193 metagenomes collated by Keller *et al.* [9], whose fuzzy-clustering approach suggested three Enterotypes as optimal cluster number. Using cvaNMF, we identified five signatures from the same data, including signature S1 (Esch/Bifi) which lacked a corresponding ET, but whose existence we know from the Enterosignature [17] approach. This signature is often a minor contributor to the overall composition in adult samples, but its importance lies in detecting dysbiotic microbiomes, as it was more often associated with disease conditions than any other signature. This is further exemplified by the ‘dysbiosis score’ that Keller *et al.* developed to estimate the uncertainty of ET assignment, identifying samples which fall somewhere on the gradient between clusters; we found a significant positive correlation between dysbiosis and the weight of S1 (Esch/Bifi) and similarly a negative correlation between our ‘model fit’ and ‘dysbiosis score’, as predicted in the Enterosignature model [17]. Changes in these often low-abundance taxa seem to explain a large proportion of gut metagenomes which cannot be assigned to the adult-derived ETs, showing the importance of identifying patterns for non-dominant groups of taxa in the human gut. We compared the machine learning models based on these five enterosignatures to Wu *et al.* who identified 141 species commonly associated with disease, and trained machine learning models from these [15]. Demonstrating the power NMF can achieve in a very low dimensional space only five enterosignatures and model fit achieved a similar or better performance for three out of six diseases, compared to a model that used 141 species abundances.

Identifying bacterial guilds and the ecosystem patterns governing these is important beyond the human gut. Therefore we used cvaNMF to analyse the microbiomes of plant root tissue and surrounding soils, as well as a global analyses incorporating multiple fungi and bacteria and including numerous hosts and environmental microbiomes. This analysis demonstrated effective stratification of important guilds, while maintaining a high level of sensitivity. Fungi and bacteria showed weakly correlated patterns of distribution using NMF decompositions—challenging to identify using discrete clustering methods—subsequently allowing us to identify discrete bacterial communities associating with environmental, host-associated external as well as internal niches, and mixtures of these in wastewater. The root and soil analysis allowed us to reassert key insights identified by Zhou *et al.* [35] using hundreds of bacterial species, including a geographic gradient in microbiome composition based on five signatures, for better distinction here named ‘terrasignatures’.

We chose to conduct our microbiome analyses at genus or class level as a balance between generalizability and functional specificity. Bacterial life strategies, ecological preferences, and functional traits can be shared at taxonomic levels as high as class or phylum [63, 64], and while specific species or strain level effects may be obscured, structure related to these important conserved traits can still be captured using data at these higher taxonomic levels. The relative abundance of taxa used as input for these microbiome analyses is compositional in nature. A limitation in common with other decomposition approaches such as unmodified PCA or LDA, NMF does not address the statistical issues inherent to compositional data, and common transformations such log-ratio transforms violate the non-negativity constraint or introduce challenges in interpreting the resulting model.

Last, our reanalysis of single cell sequencing dataset [37] intuitively separated samples based on cancer and disease state, while informing on cell populations important in either type. Salcher *et al.* found four clusters in the data, that were characterized by different kinds of immune cell infiltration: deserted, B-cells, T-cells, or myeloid macrophage/monocytes. Our three identified signatures share similarity with these clusters, with S1 containing myeloid cells and having a high weight in a subset of early stage tumours, S2 containing both B- and T-cells and being generally more abundant among early stage tumours, and S3 containing malignant cells and being dominant in late stage tumours. Salcher *et al.* found squamous cell carcinoma patient over-represented in the immune-deserted cluster; we also find the cancerous cell signature S3 significantly higher in these samples. As well as confirming these disease stage associated cell types, our signatures also described an additional difference between non-cancerous samples from those with and without COPD, identifying an increase in the T- and B-cell containing signature S2.

## Conclusion

Our software cvaNMF provides easy to use domain independent methods for rank selection, decomposition, analysis, and visualization with command line tools or as a python package, as well as scaling to large inputs through a pipeline ready to use on HPC or cloud computing systems. We have shown that NMF can provide a decomposition of large or complex data into a small number of ‘signatures’ with biologically interpretable meaning. In contrast to discrete clustering approaches, NMF decompositions describe samples as a mixture, allowing gradients in relative abundance as well as describing minor signatures in community which can be strongly associated with disease or relevant phenotypes.

## Supplementary Material

gkaf1388_Supplemental_File

## Data Availability

cvaNMF is available at https://github.com/apduncan/cvanmf and installable from pip and bioconda as cvanmf. A Nextflow pipeline is available from the same repository. A copy of the repository has been desposited on figshare (10.6084/m9.figshare.29195696). Benchmarking was carried out using pipeline https://github.com/apduncan/cvanmf_benchmark. Analysis code and input data are available in the repository https://github.com/apduncan/cvanmf_analysis, as well as deposited on figshare (10.6084/m9.figshare.28590875). Analysis of the NSCLC atlas is also shown in the cvaNMF documentation at https://cvanmf.readthedocs.io. Gut signature analysis used the Keller *et al.* [9] genus level table available from https://github.com/grp-bork/enterotypes, the original enterosignatures compositions and definitions were obtained from Frioux *et al.* [17]. Bacterial and fungal class level abundance for multiple biomes from Bahram *et al.* [36]. Soil and root microbiome composition from Zhou *et al.* [35] was summed to genus level. Cell type compositions for the NSCLC atlas was derived from data in Salcher *et al.* [37].
