# Supplementary material for: Detecting signatures underlying the composition of biological data
Source: Nucleic Acids Res. 2025 Dec 29;53(22):gkaf1388. doi: 10.1093/nar/gkaf1388 (PMC12746108; doi:10.1093/nar/gkaf1388)
Supplement: gkaf1388_Supplemental_File [file gkaf1388_supplemental_file.docx]

## Supplementary Material

## Supplementary Figures

Supplementary Figure 1: Example synthetic data used for benchmarking, for k=4. All matrices have been scaled for visualisation to $ln(1+x)$ where $x$ is the matrix. Unscaled matrices are used during benchmarking.


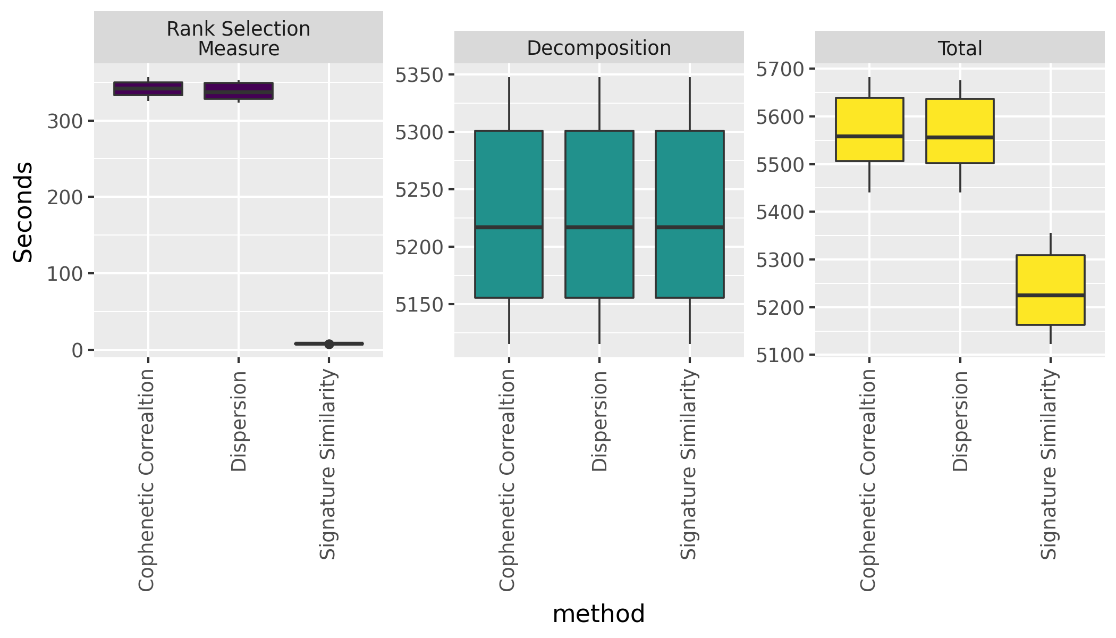


Supplementary Figure 2: Time benchmarks for stability-based rank selection methods. Ranks 2 to 15 were search for a 300 1500 matrix, repeated 10 times. Signature Similarity is an order of magnitude quicker to calculate, taking approximately 7 seconds to over 5 minutes for Cophenetic Correlation and Dispersion. All methods are based on assessing decompositions from multiple random initializations (here 100), so the time taken for decompositions is the same for all method as the same decompositions were used. The total time taken, including the decomposition and calculation of rank selection measures, is shown in the final panel.


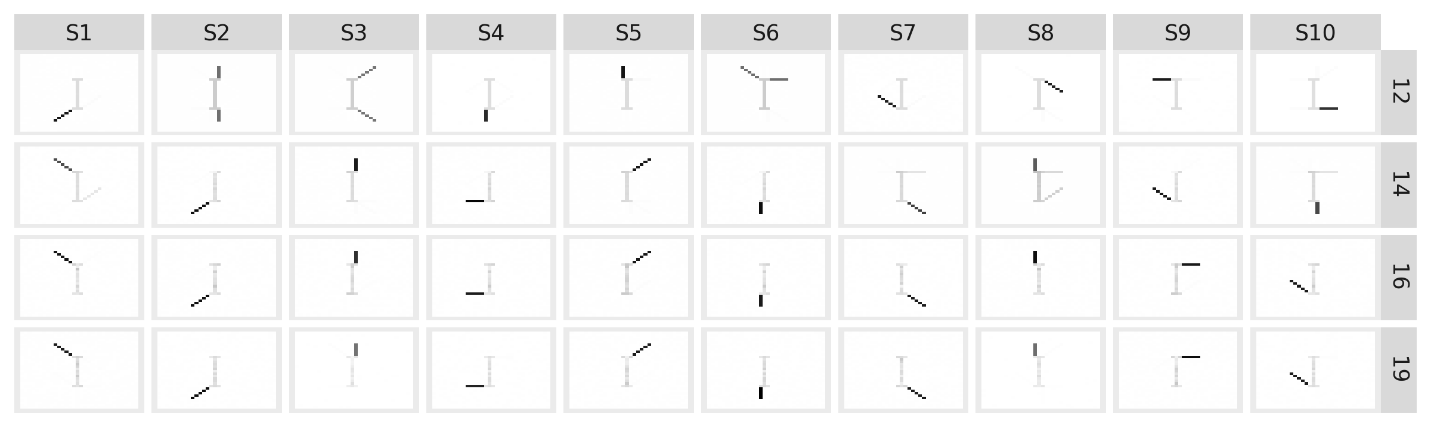

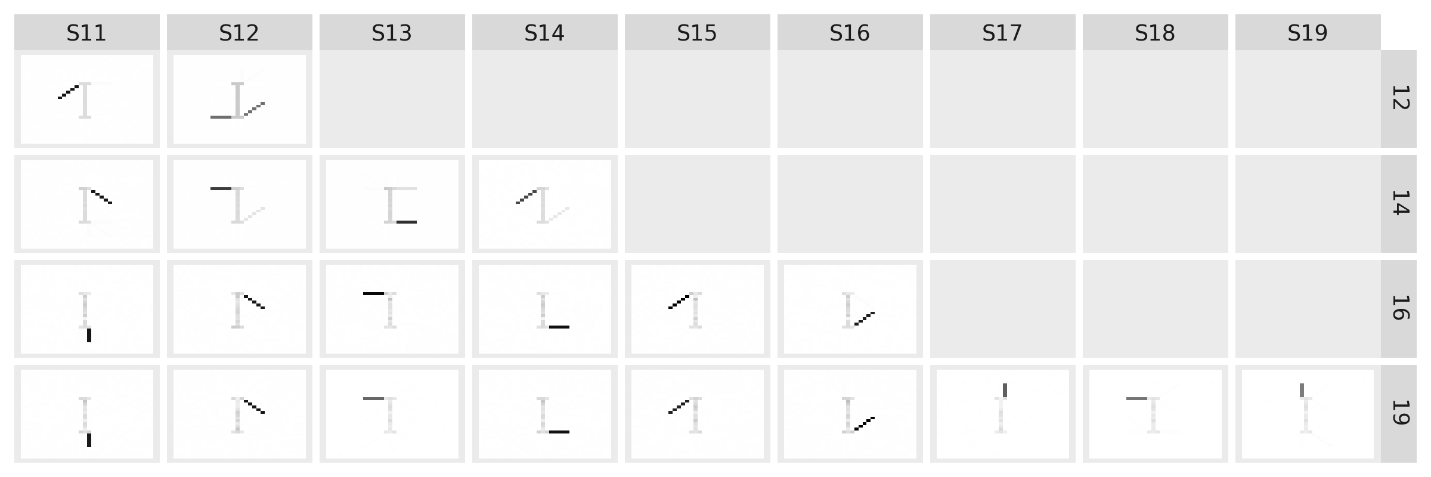


Supplementary Figure 3: Decompositions of the Swimmer data at ranks suggested by bicross-validation (k=16), dispersion (k=12), and cophenetic correlation (k=14), and too high a rank (k=19). Each row represents the signatures from a single decomposition. At rank 16, as suggested by bicross-validation, each signature contains one clear limb. In too low a rank as suggested by the dispersion coefficient multiple limbs are grouped into the same signature ( at rank 12, signatures S2, S3, S6, and S12). Too high a rank leads to limbs being split across multiple signatures (at rank 19, S13 and S18 represent the same position for example).


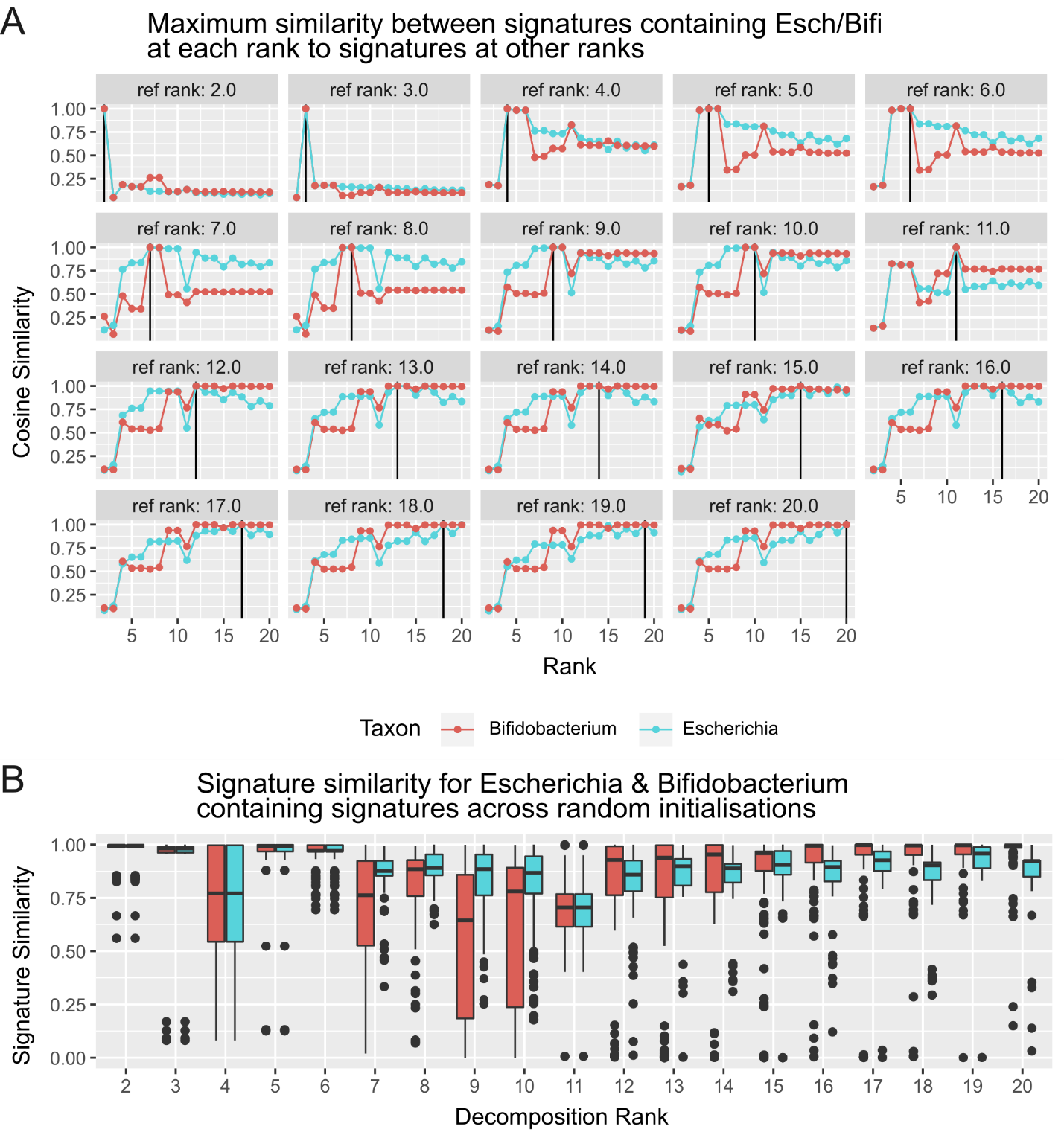


Supplementary Figure 4: Consistency of signatures containing Escherichia and Bifidobacterium, which are found in the same signature from rank 4 to rank 6, and are consistently identified across random initialisations at ranks 5 and 6. A) Each panel shows the similarity of the signatures containing Escherichia and Bifidobacterium at a given rank (indicated in panel title, and by vertical line) to the signatures containing the same taxon at different ranks. At some ranks the two are in the same signature, so only a single point appears. From ranks 4 to 6, the signatures containing Esch/Bifi are highly similar. B) Similarity of signatures containing Escherichia or Bifidobacterium from different random initialisations at the same rank, indicating how consistently the signature is recovered.


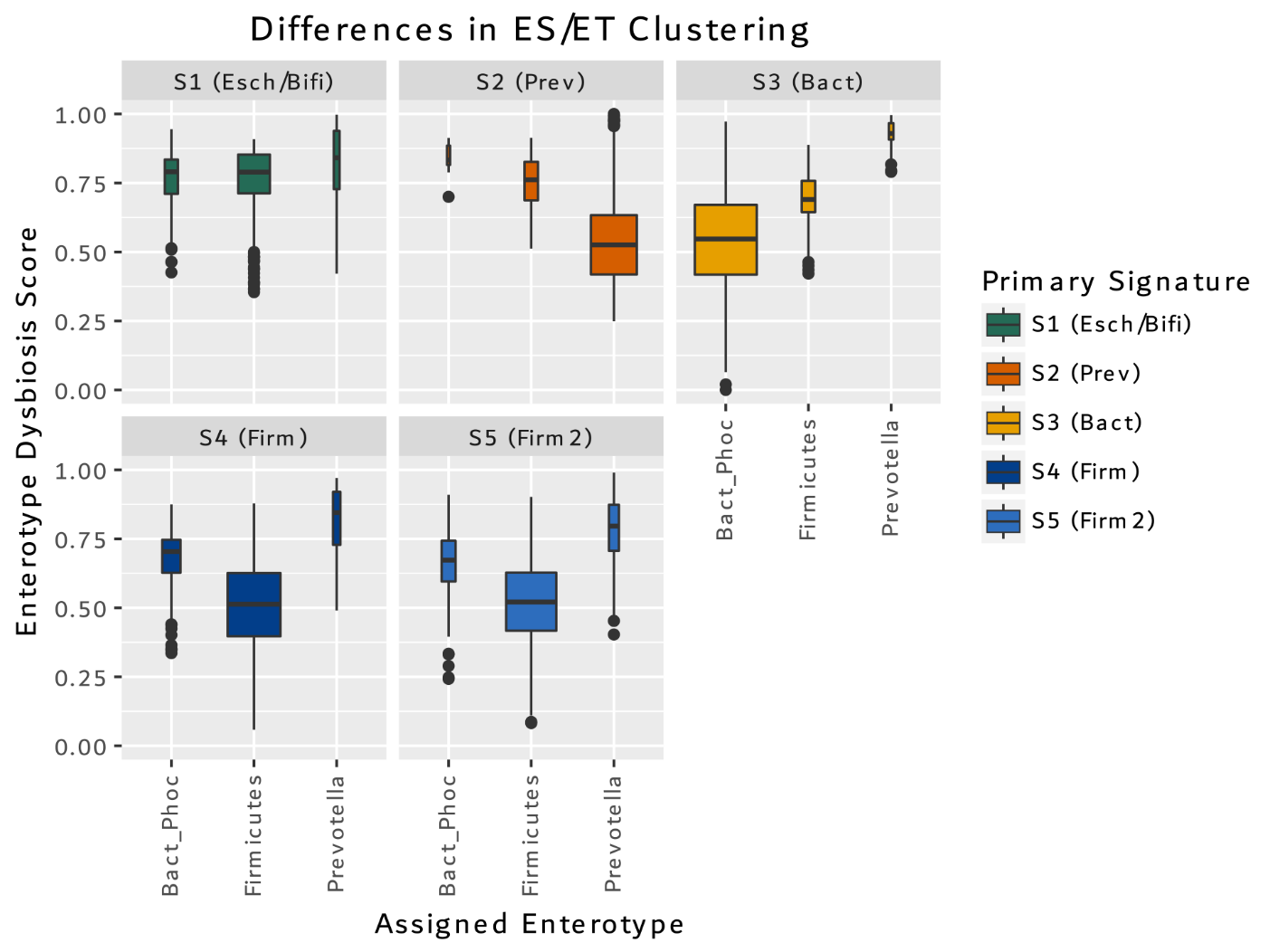


Supplementary Figure 5: Consistency of Enterotype and Signature assignment. Samples were assigned to the signature for which they had highest weight. Each panel is the samples assigned to a given Signature, and each box those from a different ET, with the width scaled to the number of samples. The ET dysbiosis score is plotted on the vertical axis.


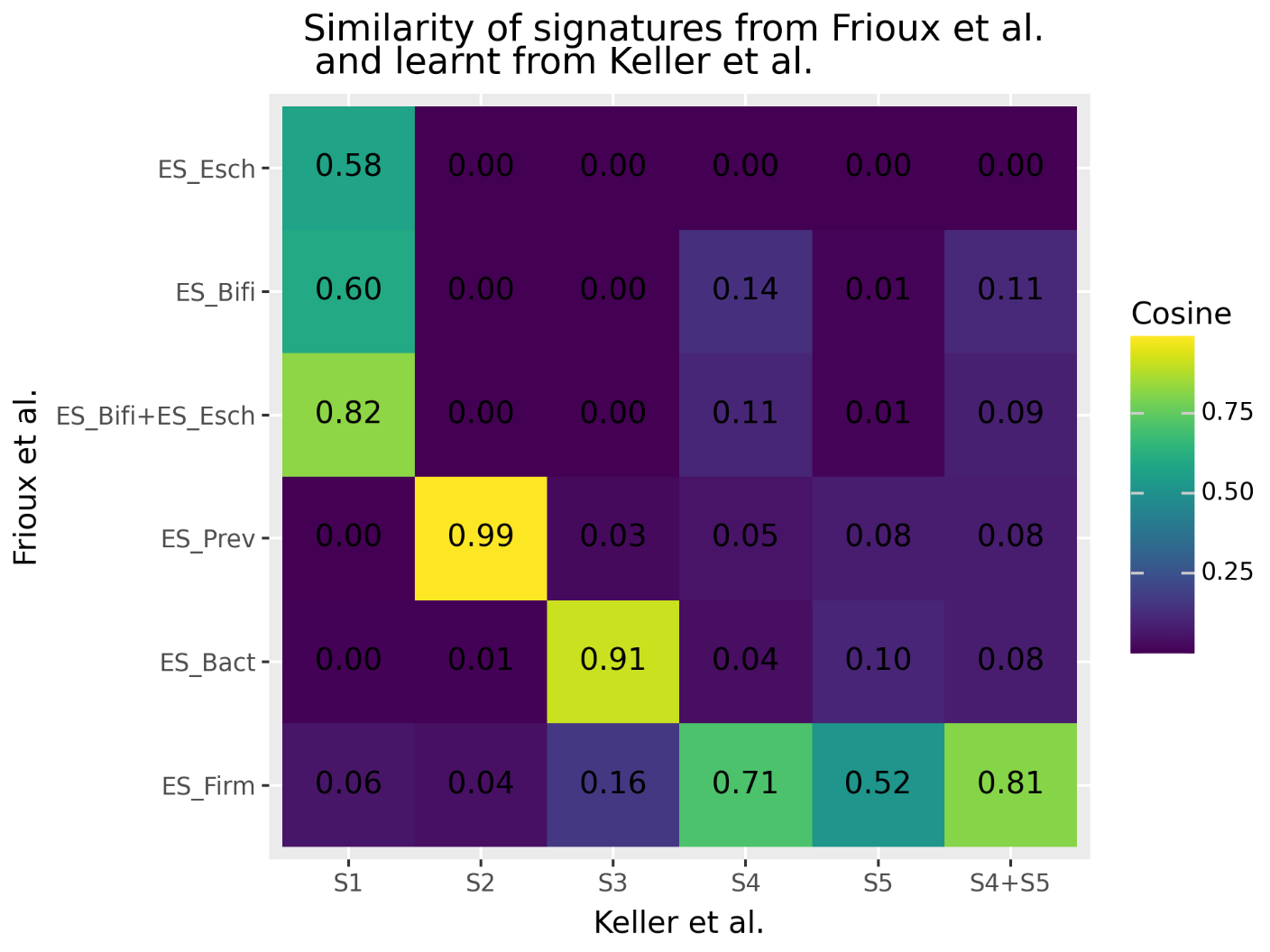


Supplementary Figure 6: Cosine similarity between signatures from Frioux et al. and Keller et al. Frioux et al. was processed using the genome-resolved pipeline MG-TK and GTDBr207, and Keller et al. mOTUs and GTDB r202. Sums of signatures S4+S5 in Keller et al. and ES_Bifi+ES_Esch were included in the comparison as they appeared to be combined in the other model.

###
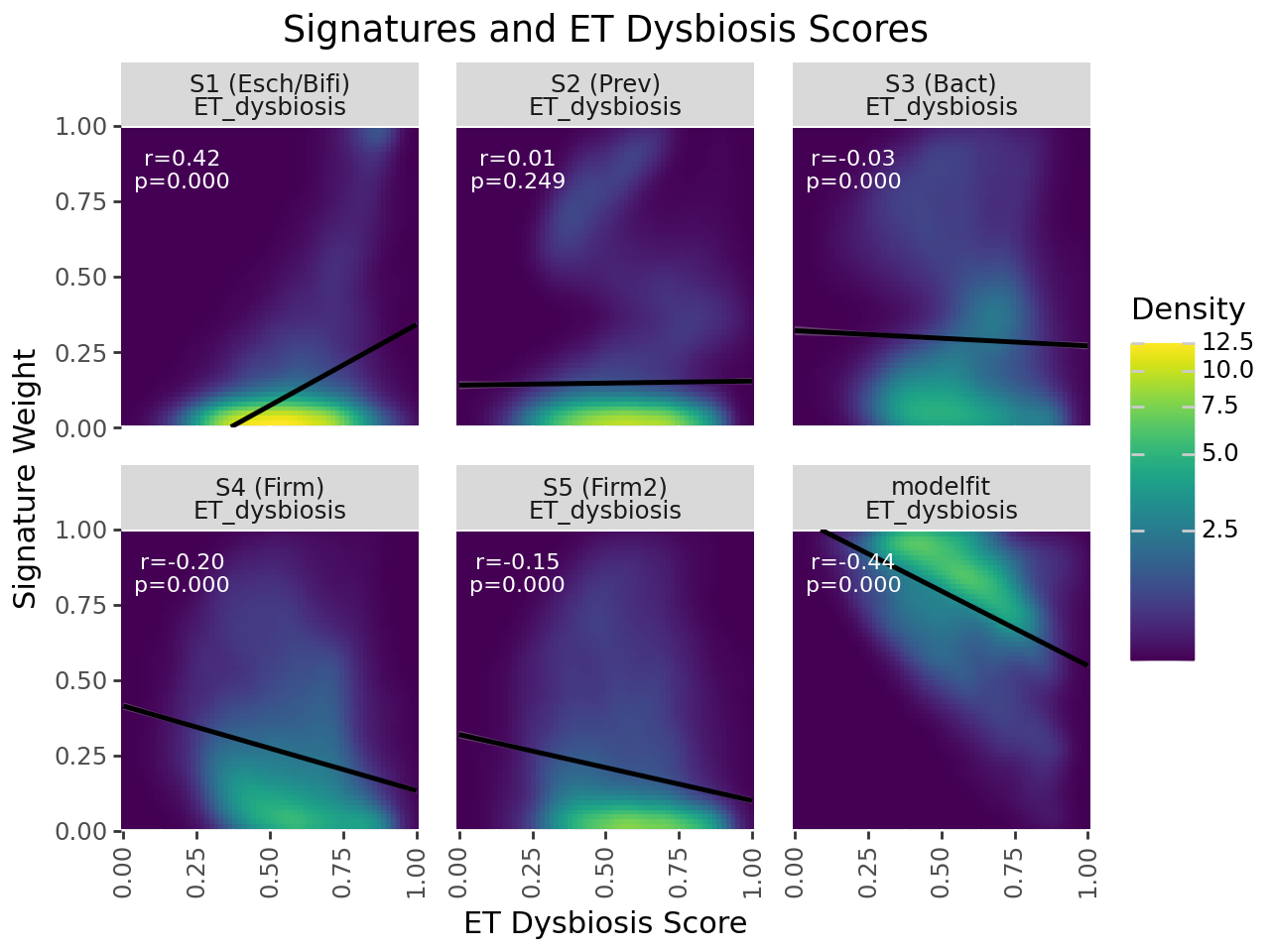


Supplementary Figure 7: Relationship between signature weights and model fit on the vertical axis to Enterotype dysbiosis score on the horizontal axis. Color indicates number of samples which fall into an area on the plot, with brighter colors indicating more samples. Color progression has been transformed to a non-linear progression as indicated in the color bar scale.


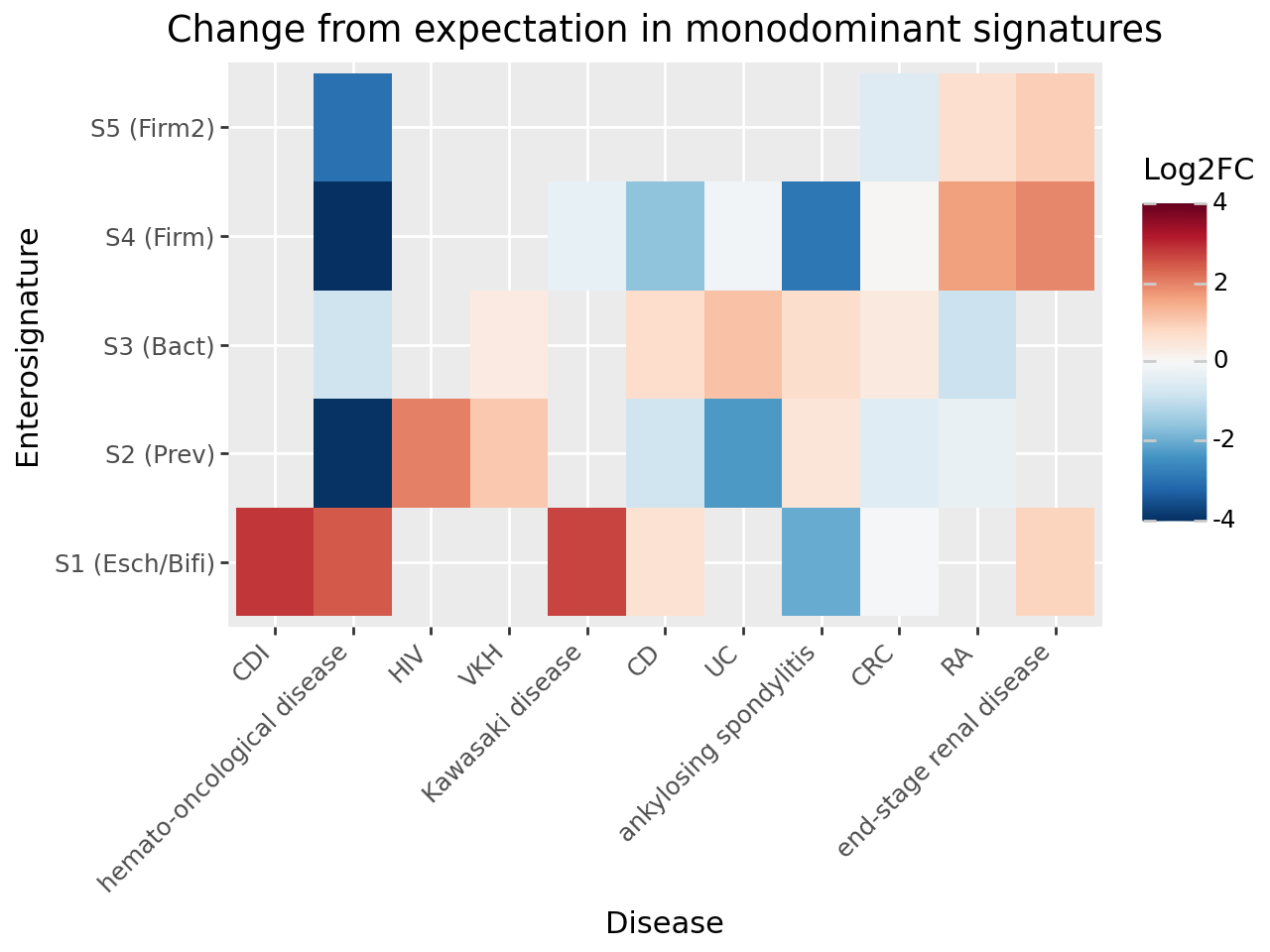


Supplementary Figure 8: Log2 fold change in observation of monodominance of each signature in comparison to samples with no disease reported. Red indicates an increase in monodominance, and blue a decrease.


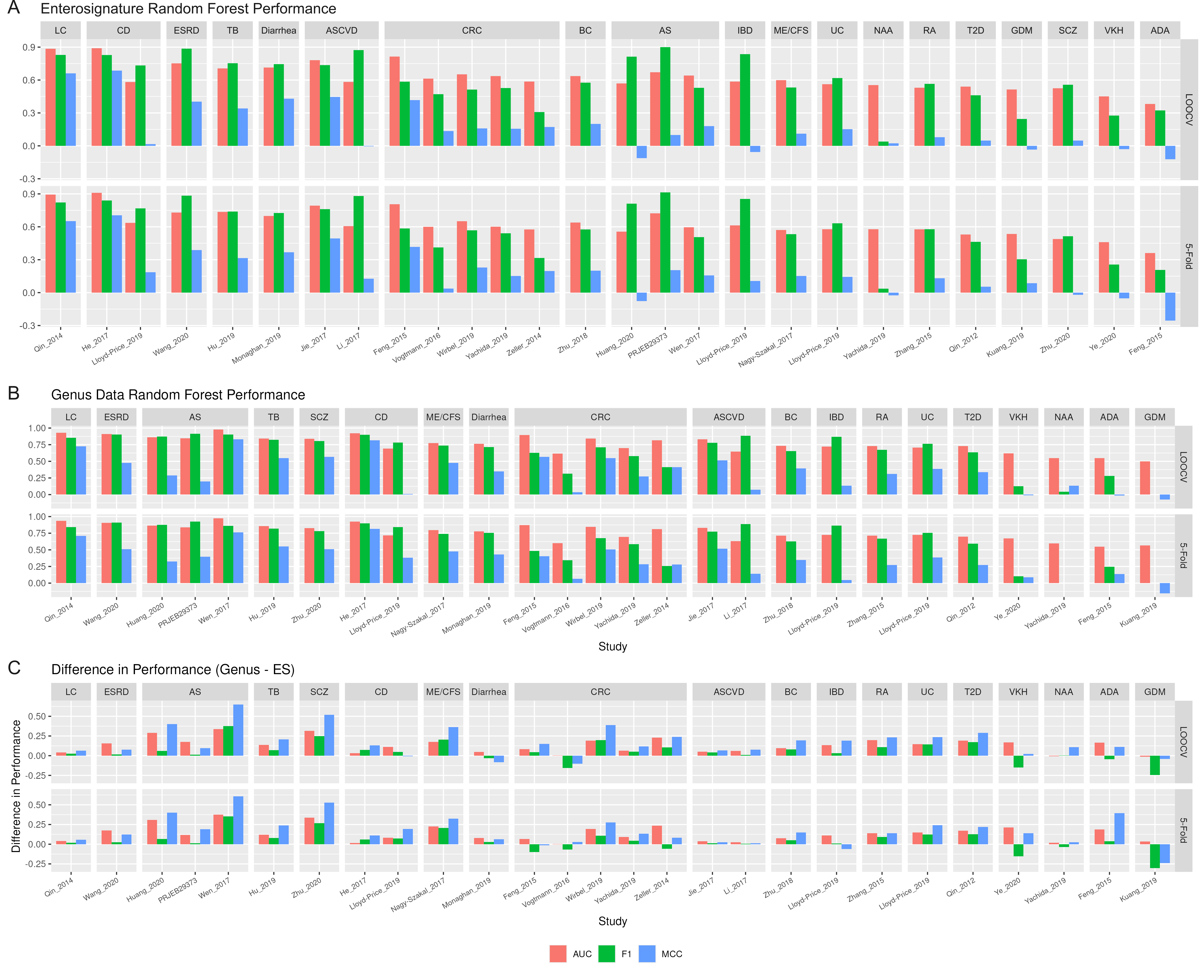


Supplementary Figure 9: A) Performance for all random forest models using Enterosignature weight, using both Leave-One-Out (top, LOOCV) and 5-Fold stratified crossvalidation. Performance is measured with ROC-AUC, F1 Score, and Matthews Correlation Coefficient (MCC). Diseases are ordered by descending mean AUC. Study labels have had the disease name truncated. B) Performance for random forest models using Genus level compositions as input C) The difference between performance for genus and Enterosignature models. Above 0 indicates greater performance for the genus level models.


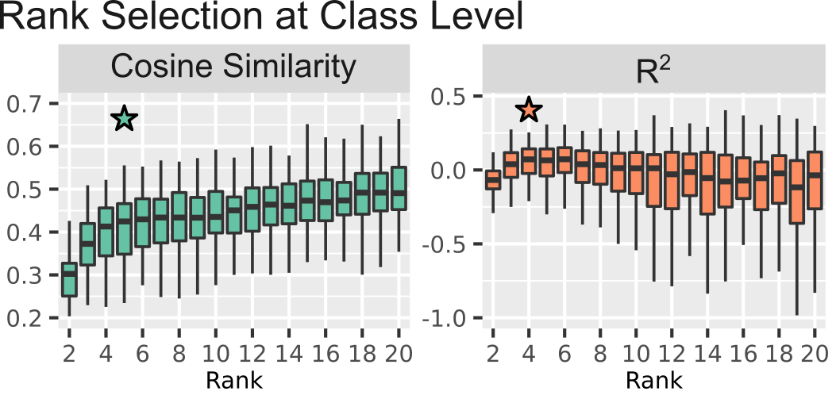


Supplementary Figure 10: Bicrossvalidation rank selection for 13,491 samples from different biomes. Stars represent ranks suggested by automated heuristics.


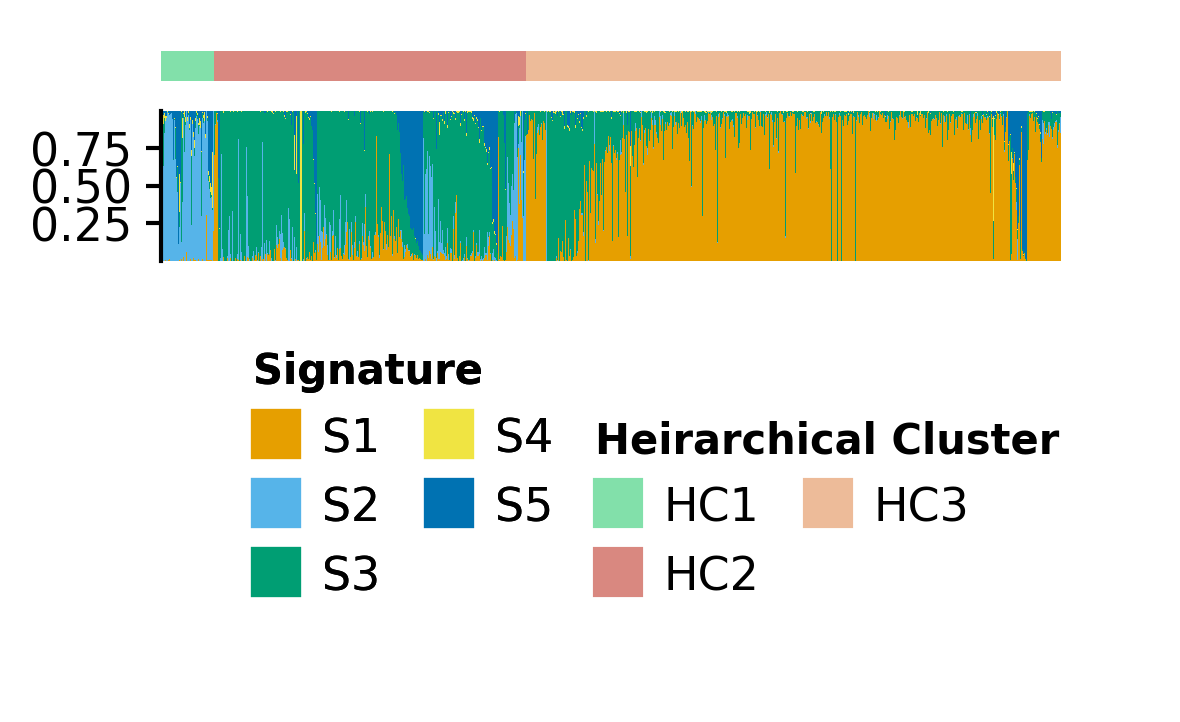


Supplementary Figure 11: Relative weight of signatures learnt from decomposition of global diversity data, with their membership in earlier hierarchical clustering indicated by the ribbon at the top. The bacterial signatures largely recapture a similar structure, with S1 high in cluster HC3, S3 high in cluster HC2, and S2 high in cluster HC1.


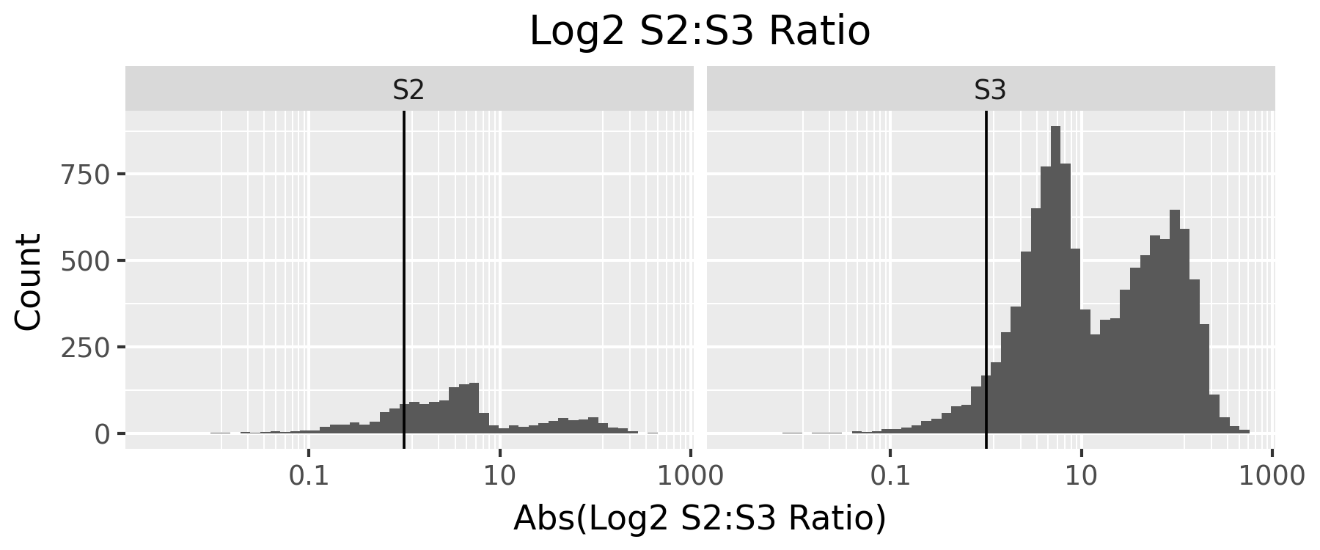


Supplementary Figure 12: Ratio of S2:S3 non-gut associated bacterial signatures. Absolute value of log2 transformed ratio is shown, with samples with S2 as a primary signature in the left panel, and S3 in the right. Vertical line is drawn at 1.0.


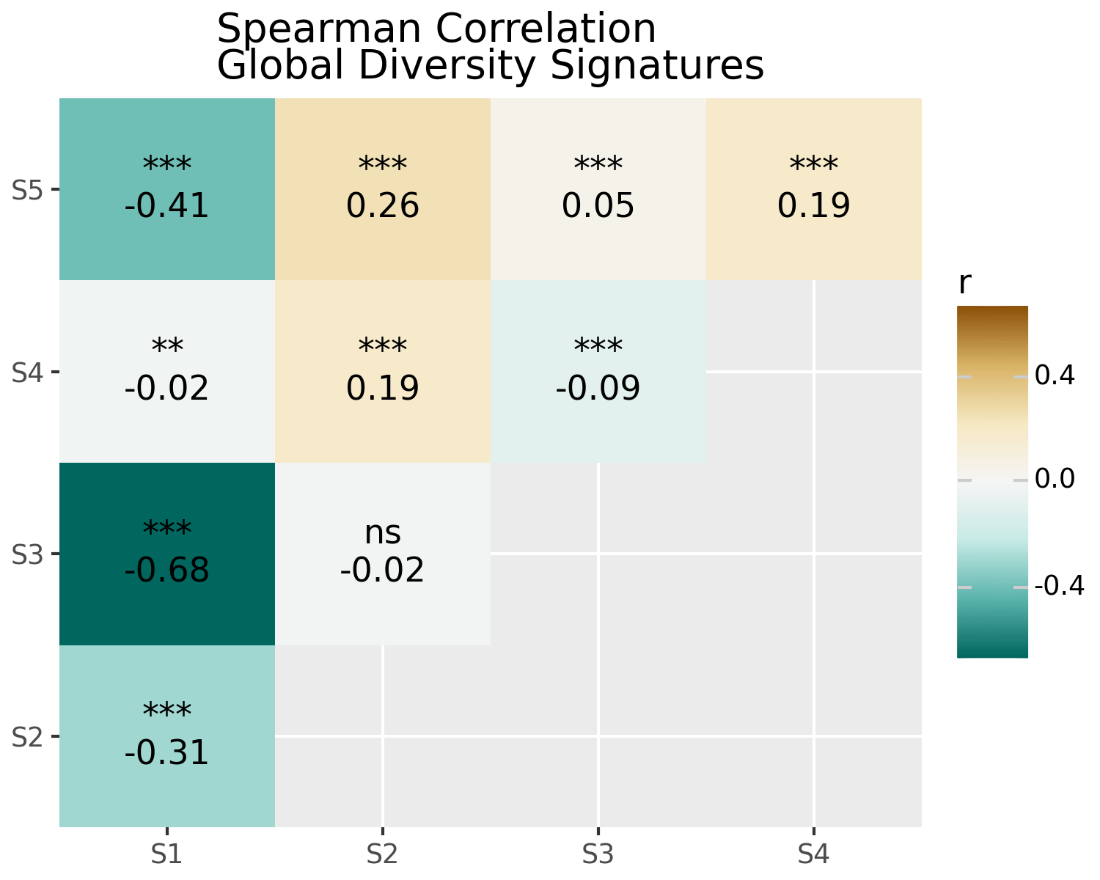


Supplementary Figure 13: Spearman correlation between signatures learnt from global diversity data. Value in cell is the correlation, p values adjust using Bonferroni-Holm method. Adjusted p values indicated by: *** p<=0.001, ** p<=0.01, * <= 0.05.


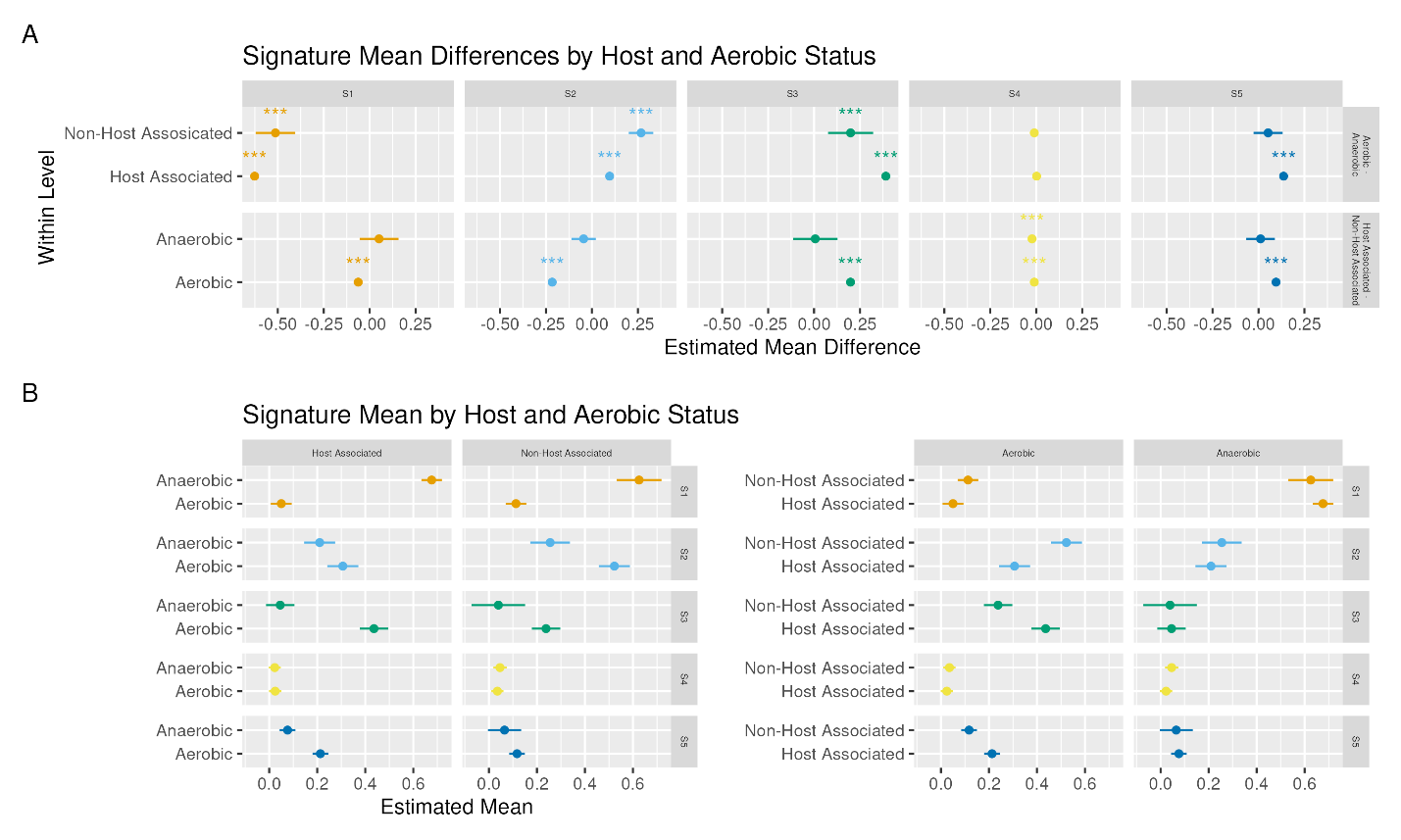


Supplementary Figure 14: Estimated means of signatures between samples grouped by host-association or an/aerobic state. A) Difference between estimated means within each group. Lines indicate 95% confidence intervals. Significance indicated by q<0.001 ***, q<0.01 **, q < 0.05 *. The top row of panels relates to the difference between the means of aerobic and anaerobic samples, within non-host associated and host-associated samples. There is no significant difference between anaerobic environments from host or non-host associated environments. B) Estimated mean relative signature weights for interactions of host and aerobic state. Left panels show that bacterial signatures S1-S3 diverge in weight between aerobic and anaerobic environments regardless of host-association. The right panel shows that relative weights are similar for aerobic or non-aerobic environments regardless of host association.


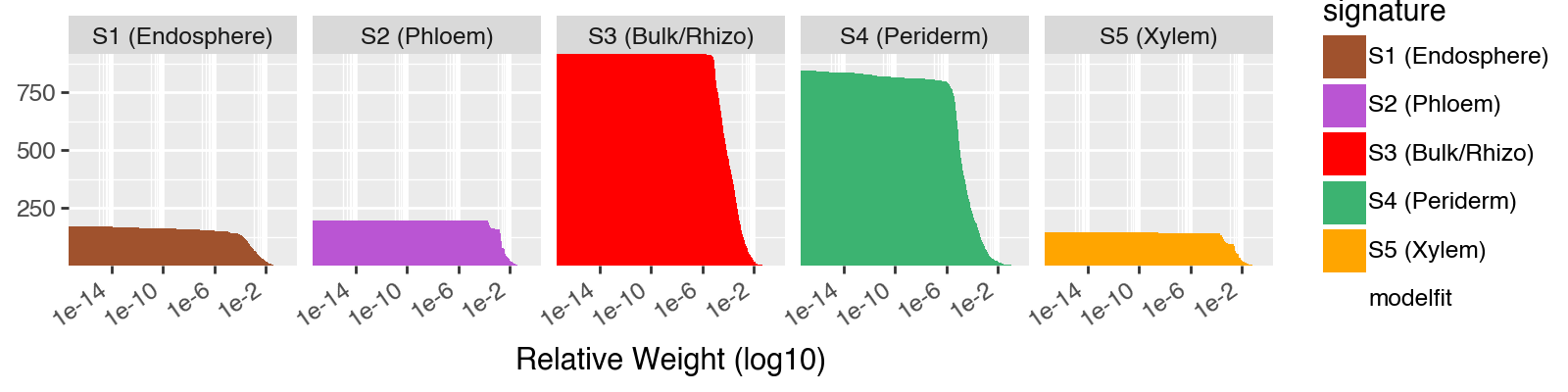


Supplementary Figure 15: Distribution of signature weights in root and soil data decomposition. Relative weights in each signature were ordered, with weight plotted on a log sale on the horizontal axis, and in descending order along the vertical axis.


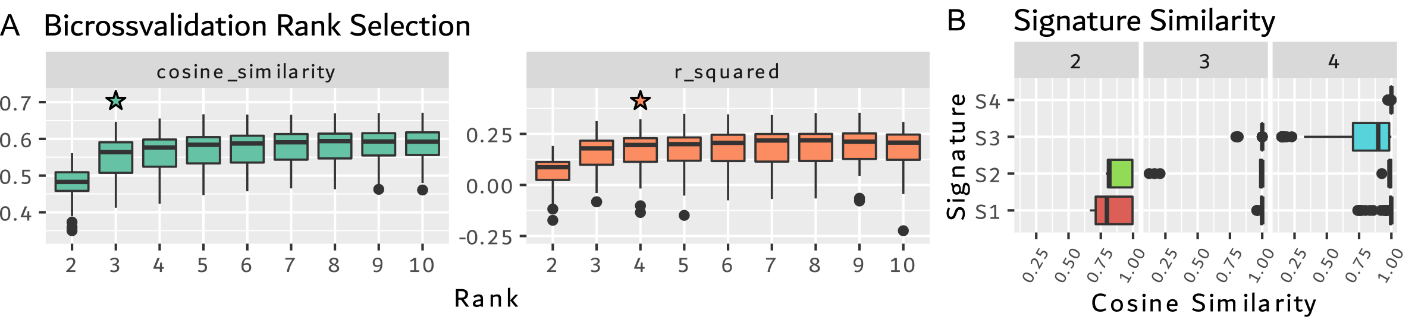


Supplementary Figure 16:A) Bicrossvalidation rank selection for NSCLC lung cancer samples. Stars indicated ranks suggested by automated heuristics. B) Signature similarity for models at ranks 2, 3, and 4. Median stability is higher at rank 3 than either 2 or 4.

Supplementary Figure 17: Schematic of bicrossvalidation. A) Input matrix is divided into blocks. B) Blocks are joined to four matrices. This is repeated with each fold in the place of A. C) $W_{B}$ and $H_{C}$ are estimated through decomposition of the other matrices, allowing an estimate $A'$ of held out block $A$ as $A^{'}=$ $W_{B}H_{C}$. Numbers refer to steps in supplementary text.


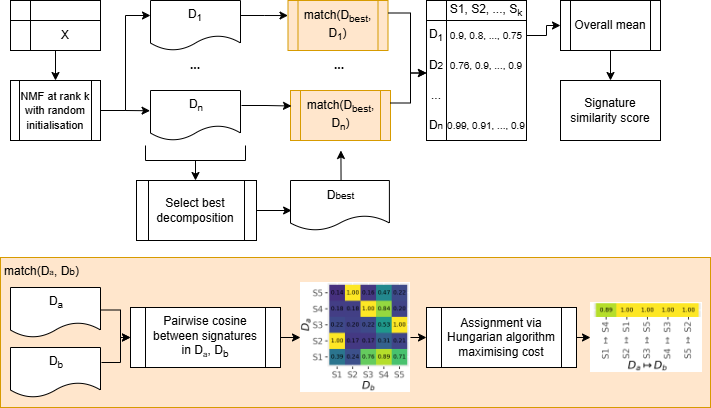


Supplementary Figure 18: Diagram showing how signature similarity score is calculated. Di is decomposition i of input X with a random initialization. Peach box at the bottom shows the match function which maps from signatures in Da to signatures in Db, and returns the cosine similarity between the matched signatures. Some example data is shown in this function for intermediate and output values.

## Supplementary Tables

|  |  | Leave-One-Out | | | Fold | | |
| --- | --- | --- | --- | --- | --- | --- | --- |
| Disease | **Studies** | **AUC** | **MCC** | **F1** | **AUC** | **MCC** | **F1** |
| CD | He_2017_Crohn | 0.89 | 0.686 | 0.828 | 0.91 | 0.705 | 0.84 |
| LC | Qin_2014_liver_cirrhosis | 0.885 | 0.661 | 0.829 | 0.894 | 0.652 | 0.822 |
| CRC | Feng_2015_CRC_Austria | 0.814 | 0.417 | 0.585 | 0.806 | 0.417 | 0.585 |
| ASCVD | Jie_2017_artherosclerosis_cardiovascular | 0.781 | 0.446 | 0.736 | 0.793 | 0.494 | 0.761 |
| ESRD | Wang_2020_renal_disease | 0.752 | 0.403 | 0.886 | 0.73 | 0.388 | 0.884 |
| Diarrhea | Monaghan_2019_India | 0.714 | 0.43 | 0.745 | 0.699 | 0.368 | 0.726 |
| TB | Hu_2019_tubercolosis | 0.706 | 0.341 | 0.753 | 0.736 | 0.315 | 0.739 |
| AS | PRJEB29373_ankylosing_spondylitis | 0.671 | 0.099 | 0.9 | 0.723 | 0.205 | 0.914 |
| CRC | Wirbel_2019_CRC_Germany | 0.652 | 0.159 | 0.513 | 0.651 | 0.229 | 0.568 |
| AS | Wen_2017_ankylosing_spondylitis | 0.641 | 0.18 | 0.529 | 0.596 | 0.157 | 0.506 |
| CRC | Yachida_2019_CRC | 0.636 | 0.156 | 0.527 | 0.601 | 0.152 | 0.541 |
| BC | Zhu_2018_breastcancer | 0.636 | 0.2 | 0.576 | 0.639 | 0.2 | 0.576 |
| CRC | Vogtmann_2016_CRC_USA | 0.613 | 0.135 | 0.471 | 0.6 | 0.037 | 0.412 |
| ME/CFS | Nagy-Szakal_2017_CFS | 0.599 | 0.111 | 0.532 | 0.571 | 0.152 | 0.533 |
| CRC | Zeller_2014_CRC_France | 0.586 | 0.172 | 0.308 | 0.576 | 0.197 | 0.316 |
| IBD | Lloyd-Price_2019_HMP2IBD | 0.586 | -0.056 | 0.836 | 0.613 | 0.106 | 0.854 |
| ASCVD | Li_2017_hypertension | 0.583 | -0.004 | 0.873 | 0.606 | 0.127 | 0.881 |
| CD | Lloyd-Price_2019_HMP2IBD | 0.582 | 0.016 | 0.733 | 0.636 | 0.186 | 0.768 |
| AS | Huang_2020_ankylosing_spondylitis | 0.569 | -0.112 | 0.813 | 0.556 | -0.077 | 0.811 |
| UC | Lloyd-Price_2019_HMP2IBD | 0.562 | 0.152 | 0.618 | 0.578 | 0.144 | 0.632 |
| NAA | Yachida_2019_CRC | 0.554 | 0.023 | 0.039 | 0.578 | -0.025 | 0.036 |
| T2D | Qin_2012_T2D | 0.54 | 0.048 | 0.461 | 0.53 | 0.054 | 0.463 |
| RA | Zhang_2015_rheumatoid_arthritis | 0.53 | 0.079 | 0.565 | 0.577 | 0.131 | 0.578 |
| SCZ | Zhu_2020_schizophrenia | 0.525 | 0.048 | 0.557 | 0.489 | -0.02 | 0.514 |
| GDM | Kuang_2019_gestational_diabetes | 0.514 | -0.034 | 0.245 | 0.535 | 0.086 | 0.304 |
| VKH | Ye_2020_Vogt-Koyanagi-Harada | 0.451 | -0.03 | 0.276 | 0.46 | -0.052 | 0.256 |
| ADA | Feng_2015_CRC_Austria | 0.382 | -0.122 | 0.323 | 0.361 | -0.255 | 0.207 |

Supplementary Table 1: random forest performance using Leave-One-Out Crossvalidation and 5-Fold Crossvalidation in predicting disease from signature weight and model fit, based on data from Keller et al. [9]. Study column indicates which study the model was trained using, and disease the disease from the study being classified. MCC = Matthew’s Correlation Coefficient.
